# Crowdsourced Identification of Potential Target Genes for CTV Induced Gene Silencing for Controlling the Citrus Greening Vector *Diaphorina citri*

**DOI:** 10.3389/fphys.2021.571826

**Published:** 2021-04-09

**Authors:** John E. Ramos, Ritesh G. Jain, Charles A. Powell, William O. Dawson, Siddarame Gowda, Dov Borovsky, Robert G. Shatters

**Affiliations:** ^1^U.S. Horticultural Research Laboratory (USDA-ARS), Fort Pierce, FL, United States; ^2^Indian River Research and Education Center, UF/IFAS, Fort Pierce, FL, United States; ^3^Citrus Research and Education Center, UF/IFAS, Lake Alfred, FL, United States; ^4^Department of Biochemistry and Molecular Genetics, University of Colorado School of Medicine, Aurora, CO, United States

**Keywords:** *Diaphorina citri*, RNA interference, *Citrus tristeza virus* engineering, gene silencing with dsRNA, Rieske down regulation

## Abstract

Citrus Greening or Huanglongbing (HLB) is a disease of citrus, causing high reduction in citrus production and is transmitted by the Asian citrus psyllid *Diaphorina citri* Kuwayama vectoring a phloem-limited bacterium *Candidatus Liberibacter* sp. We report research results using crowdsourcing challenge strategy identifying potential gene targets in *D. citri* to control the insect using RNA interference (RNAi). From 63 submitted sequences, 43 were selected and tested by feeding them to *D. citri* using artificial diet assays. After feeding on artificial diet, the three most effective dsRNAs causing 30% mortality above control silenced genes expressing iron-sulfur cluster subunit of the mitochondrial electron transport chain complex (Rieske), heme iron-binding terminal oxidase enzyme (Cytochrome P450) and tetrahydrobiopterin (BH_4_) pathway enzyme (Pterin 4α-Carbinolamine Dehydratase). These sequences were cloned into a citrus phloem-limited virus (*Citrus tristeza virus*, CTV T36) expressing dsRNA against these target genes in citrus. The use of a viral mediated “para-transgenic” citrus plant system caused higher mortality to adult *D. citri* than what was observed using artificial diet, reaching 100% when detached citrus leaves with the engineered CTV expressing dsRNA were fed to adult *D. citri*. Using this approach, a virus-induced gene silencing (VIGS) can be used to test future transgenic cultivars before genetically engineering citrus. RNA Seq analysis after feeding *D. citri* CTV-RIE on infected leaves identified transcriptionally modified genes located upstream and downstream of the targeted RIE gene. These genes were annotated showing that many are associated with the primary function of the Rieske gene that was targeted by VIGS.

## Introduction

Citrus Greening or Huanglongbing (HLB) is a disease of citrus, first discovered in the state of Florida in 2005 (Stokstad, 2012), that is responsible for a greater than fifty percent reduction in citrus production and is now present in other citrus producing states (National Agricultural and Statistics Service, 2005; Putnman et al., 2011). *D. citri* (*Diaphorina citri* Kuwayama) is the only known insect vector of the HLB associated phloem-limited bacterium *Candidatus Liberibacter* sp. In spite of aggressive approach to control *D. citri*, the disease has continued to spread in Florida and there is concern over the loss of pesticide effectiveness and residue accumulation in the fruit and in the environment (Ichinose et al., 2010; Tiwari et al., 2011). In an effort to develop a biological pest control strategy a global crowd sourcing challenge was issued by the United States citrus industry to identify the best *D. citri* transcript target(s) for RNA interference (RNAi) control strategy. RNAi is a eukaryotic genetic defense strategy that evolved in order to confer primitive immunity to infection (Covey et al., 1997; Qiu et al., 2005; Ben Berkhout, 2006; Marques et al., 2006; Wang et al., 2006; Maillard et al., 2013). dsRNAs from Viral RNA genomes and foreign transcripts are recognized by RNase III (an endoribonuclease) that clips dsRNA molecules into small (20–25 base) fragments, or small interfering RNAs (siRNAs). A single siRNA strand is then incorporated into the RNA Induced Silencing Complex (RISC), which uses the siRNA to bind (by hybridization) mRNAs in the cytosol by sequence complementary recognition. The catalytic subunit of the RISC (a member of the Argonaut protein family with endonuclease activity) then cleaves the mRNA rendering it non-functional (Bernstein et al., 2001; Lipardi et al., 2001). This effect can be redirected against the host cell by the exogenous introduction of dsRNAs having sequence identity for host genes, resulting in the reduction of host cell mRNA abundance and subsequent loss of function (Fire et al., 1998; Gregory et al., 2005). RNAi silencing of host genes has previously been demonstrated in eukaryotic organisms (including insects) following oral uptake of synthetic dsRNA molecules (Velásquez et al., 2009; Borgio, 2010; Gong et al., 2011; Pitino et al., 2011; Wuriyanghan et al., 2011; Galdeano et al., 2017; Yu et al., 2017; Yu and Killiny, 2018, 2020; Lu et al., 2019).

Preliminary reports suggest that *D. citri* and *Bactericera cockerelli* (potato/tomato psyllid) are vulnerable to dsRNA induced RNAi toxicity (Wuriyanghan et al., 2011; El-Shesheny et al., 2013; Galdeano et al., 2017; Yu et al., 2017; Yu and Killiny, 2018; Lu et al., 2019). These observations led to the support of the Florida Citrus industry through the Florida Research and Development Foundation (CDRF) to fund a web-based challenge through InnoCentive, Inc. Sequences that induce an RNAi-based toxicity in *D. citri* were identified by “solvers” (scientists and experts) as potential *D. citri* gene targets for silencing by oral uptake of synthetic dsRNAs using artificial diet (Hall et al., 2010). Forty-three genes were ultimately targeted and screened for susceptibility to gene silencing causing *D. citri* mortality. Of these, the Rieske gene, that expresses an iron sulfur cluster, a soluble domain of Complex III of the electron transport chain (ETC), is known to cause mortality in other insects. Therefore, proteins involved in ETC aerobic metabolism could be used as potential targets for pest control using RNA interference (Wuriyanghan et al., 2011). In addition, RNAi targeting was observed on G-protein coupled receptors with transmembrane sequences like chemokine receptors inhibiting HIV-1 replication (Martinez et al., 2002), as well as soluble proteins like juvenile hormone acid methyl transferase, causing inhibition of pupal to adult development in mosquitoes (Van Ekert et al., 2014).

Delivery of dsRNA to target insects is usually done by injection or by oral feeding using artificial diets. The latter is preferred because it does not cause injury to the target insect and was successfully demonstrated with *Aedes aegypti* larvae, Triatomine Bug (*Rhodnius prolixus*), light brown apple moth (*Epiphyas postvittana*), tsetse fly (*Glossina morsitans morsitans*), and diamondback moth (*Plutella xylostella*) (Velásquez et al., 2009; Borgio, 2010; Gong et al., 2011; Wuriyanghan et al., 2011; Van Ekert et al., 2014). Initially, dsRNA was mixed with the artificial diet that was used to feed insects. Later, long hair pin dsRNA was expressed in specific target plants to control insects (Martinez et al., 2002; Hall et al., 2010; Wuriyanghan et al., 2011; El-Shesheny et al., 2013) or in yeast cells (Van Ekert et al., 2014) to control mosquito larvae. A novel new technique using *Citrus tristeza virus* (CTV) expressing dsRNA in citrus has been recently introduced. Using this technique an abnormal wing disk (*Awd*) dsRNA has been expressed in citrus causing *D. citri* nymphs that fed on CTV infected trees to develop into adults with malformed wings (Hajeri et al., 2014). Bioassays using *in planta* approach was used by Taning et al. (2016) showed that *D. citri* is sensitive to ingested dsRNA showing a strong RNAi response prompted us to explore the potential of RNAi strategy to control the Asian citrus psyllid.

This report describes the biological activity of dsRNA molecules initially tested by feeding adult *C. citri* using artificial diet. Although many dsRNA molecules caused mortalities at a high dose, we selected only dsRNA molecules that exhibited dose response RNA interference. Because CTV is the most abundant virus in Florida citrus and is found where *D. citri* populations affect citrus (Britt et al., 2020) we engineered these molecules into attenuated CTV and expressed them in citrus leaves to protect against *D. citri*. We also report that targeting ETC membrane proteins by dsRNA in artificial diet is not as effective as targeting the soluble ETC subunit Rieske. These results suggest that soluble extra membranous domains such as Rieske or those that share similar functionality (iron-sulfur binding), are more sensitive to RNA interference (Boykin et al., 2012).

## Materials and Methods

### Experimental Insects

*Diaphorina citri* were reared on *Citrus macrophylla* plants in screen cages in an insectary at 25°C with 13:11 h light: dark cycle. Every week, 200 *D. citri* were removed to freshly flushing plants, allowed to lay eggs for 7 days and then removed from the plant leaves by aspiration. Nymphs and eggs were collected by brushing them off the flush with a small paint brush or by gently removing them with the tip of a pin. Insects that were used for RNA isolation were stored at −80°C.

### Artificial Diet Bioassay

**FIGURE 1:**
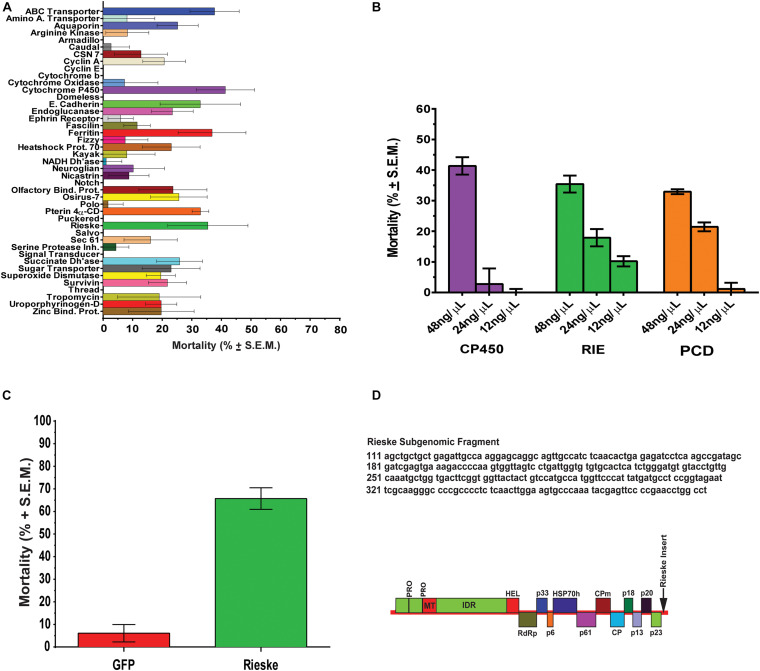
RNA interference studies. **(A)** Mortality of *D. citri* after feeding on dsRNA (48 ng/μL) of different target genes. The results are expressed as mean mortalities (%) corrected with mortalities observed by feeding on diet without dsRNA (control) of 6 determinations ± S.E.M. Twenty dsRNAs out of 43 tested caused significant mortalities above control diets. **(B)** Dose response of genes (*CP450*, *RIE*, and *PCD*) that were most significantly down regulated by a single concentration of dsRNA (48 ng/μL) in **(A)** using concentrations of 48, 24, and 12 ng/μl, respectively. **(C)** Effect of engineered CTV-*RIE* and CTV-*gfp* on *D. citri* feeding on *C. macrophylla* infected leaves using single leaf bioassays. The infected *C. macrophylla* leaves expressed *RIE* (Rieske) and *gfp* subgenomic dsRNAs of Green Fluorescent Protein of *A. victoria* GFP gene sequence that does not share sequence identity with *D. citri* genes. Mortalities are expressed as means of 6 determination ± SEM and were corrected with mortalities observed by feeding *D. citri* on leaves infected with CTV-wt. **(D)** CTV viral amplicon used to infect *C. macrophylla* is shown including the *RIE* subgenomic sequence that was cloned at the p23 locus in the 3′-NTR (non-translated region, arrow).

Bioassays were performed using 4-week-old *D. citri* (10–20 male and female adults) in a 35 mm × 10 mm Greiner Bio-One petri dishes. *D. citri*, were contained in dishes with stretched parafilm and diet containing dsRNA (200 μL) (Supplementary Table 1) was applied to the exterior-top surface of the stretched parafilm and sandwiched by a second sheet of stretched parafilm. Adult *D. citri* (10–20 per group) 1 week after adult eclosion were fed on four different concentrations of dsRNAs for 6 days. Control groups were fed artificial diets without dsRNA (Hall et al., 2010) and each feeding experiment was repeated six times. Forty-three different dsRNA (48 ng/μL) were fed to *D. citri* for 6 days and adult mortality expressed as percentage was recorded. Results were corrected for mortalities caused by diet without dsRNA and expressed as means of 6 determinations ± S.E.M. (Figure 1A). Student’s *t*-tests using *Graphpad Prism 6.0* were used for statistical analyses of the data for all the 43 dsRNA. Bioassays were performed weekly for 6 days and three gene targets were tested using dsRNA (275–325 bp) enriched diets at concentrations of 48, 24, 12, 6, and 3 ng/μL and each assay was repeated six-times. Homologous *D. citri* sequences were identified using existing genomic and transcriptomic data mining, primers were designed to amplify a short region (275–325 bp) of each gene which served as a template for dsRNAs synthesis using the MEGAscript RNAi kit (Life Technologies) (Supplementary Tables 1, 2). Diet without dsRNA was used to measure background mortality (Hall et al., 2010) that were subtracted from the mortality in each chamber. Since dsRNA of *gfp* a gene from *Aequorea victoria* that is not found in psyllids it has a very low effect on psyllid mortality (Figure 1C) testing psyllids without the addition of dsRNA to the artificial media is a good control to check for background mortality as was shown earlier by Hall et al. (2010). Feeding was stopped at day 6 because mortalities beyond day 6 often exceeded 40% when *D. citri* were fed on diet alone without dsRNA. Each experiment was repeated at least six times.

### Genetic Engineering of CTV and Inoculation of Citrus

*Citrus tristeza virus* is a single-stranded (+ sense) RNA virus that forms a dsRNA replicative genome form at p23, a 3′ replicon, and expresses variable lengths of subgenomic dsRNAs (sgRNA) (Figure 1D). The 283 bp subgenomic Rieske sequence (RIE) was cloned into pCTV9, a cDNA clone of CTV T36 (GenBank Accession number AY170468), between the p23 locus and the 3′NTR (Figure 1D) using *Pac*I and *Stu*I restriction sites (Ayllón et al., 2003; Tatineni et al., 2008).

The engineered virus was amplified by PCR and transfected into *Nicotiana benthamiana* using polyethylene glycol (Mehta et al., 2009; Sztal et al., 2012) and virions extracted from snap frozen protoplasts in 40 mM Na_2_HPO_4_ buffer pH 8.2 (Navas-Castillo et al., 1998; Satyanarayana et al., 1999). After centrifugation for 4 min at 150 *g*, the supernatant was screened for CTV by Northern blot analysis using DIG Northern Starter Kit (Roche) and Northern Max Kit (Life Technologies) and inoculated into 1 year old *C. macrophylla* trees and allowed to incubate for 6 months. After incubation, leaves were extracted and screened by RT-qPCR using primers against RIE dsRNA sequence expressed by CTV. Relative CTV titer was determined using a ratio of CTV amplification efficiency (A_*CTV*_) to citrus dehydrin (CD) gene (a ubiquitous citrus reference gene) amplification efficiency (A_*CD*_), before inoculating the genetically engineered CTV into *C. macrophylla* for virus-induced gene silencing (VIGS). An average relative CTV titer of 5.3 for this study was calculated using the equation: (A_*CTV*_^–Ct^)/(A_*CD*_^–Ct^) (Livak and Schmittgen, 2001; Yuan et al., 2006).

### Leaf Bioassay

Single and fully expanded young leaves were removed from citrus trees that were inoculated with genetically engineered CTV and the petiole placed in Eppendorf tube filled with water (1.2 mL). The tubes were sealed with parafilm and transferred into a conical tube (50 mL) containing adult *D. citri* (15–20) and the tube capped with a screened lid (El-Desouky et al., 2013). Adult *D. citri* were fed on leaves infected with engineered CTV-RIE and compared with adult *D. citri* that were fed on leaves infected with CTV-GFP.

### RNA Preparation

RNA was extracted from 10 whole adult *D. citri* or surgically removed 20 guts with TRIzol^®^ (Life Technologies, Carlsbad, CA, United States) following a modified manufacturer protocol (Chomczynski and Sacchi, 1987). Briefly, adults and guts were separately transferred into tubes containing acid-washed glass beads (180 μm) (Sigma). TRIzol was added and the samples were incubated for 5 min at room temperature. Tissues were broken in a Fast Prep instrument for 40 s at speed 6 m/s, chloroform added, and the samples incubated for 10 min at room temperature. Tissues were pelleted by centrifugation (12,000 rpm for 10 min at 4°C) and the aqueous phase transferred into a fresh tube. RNA was precipitated with isopropanol and samples were incubated at room temperature for 10 min, centrifuged (12,000 rpm for 8 min at 4°C). The pellet was washed with 75% ethanol, samples centrifuged (8,500 rpm for 5 min at 4°C) and ethanol was removed, and the RNA pellet air-dried for 10 min. The pellets were dissolved in water, and RQ1 DNase and DNase buffer (Promega, Madison, WI, United States) were added and samples incubated at 37°C for 1 h. After incubation, water, and phenol (pH 4.3) were added and the samples centrifuged (12,000 rpm for 6 min at 4°C), the upper phase transferred into a fresh tube and an equal volume of phenol/chloroform was added, samples centrifuged, and the upper phase transferred into a fresh tube and precipitated in sodium acetate (3M, pH 5.2) and 100% ethanol at −20°C overnight. The pellets were centrifuged (12,000 rpm for 15 min at 4°C), washed in 75% ice cold ethanol, re-centrifuged (12,000 rpm for 8 min at 4°C) and the ethanol removed and the pellet, air dried and suspended in water. RNA concentrations were determined at A_260_ and A_280_ in a NanoDrop 1000 instrument (Thermo Fisher Scientific, Waltham, MA, United States), and stored at −80°C. Extracted RNA was amplified using SMART^TM^ mRNA amplification kit (Clonetech) using SMART^TM^ MMLV Reverse Transcriptase. Poly G SMART^TM^ T7 extension primers, Advantage 2 polymerase mix, and RNAse H were used to create a sense cDNA template with T7 promoter. SMART^TM^ RNA polymerase was used to amplify a sense mRNA with linear amplification maintaining a relative constant cDNA copy number (Clonetech). The cDNA and mRNA were purified using NucleoSpin Gel and PCR Clean-up and NucleoSpin RNA II Purification kit (Macherey-Nagel), respectively. The concentration, nucleotide size distribution, and RNA integrity number (RIN) of the purified mRNA was determined using a 2100 Bioanalyzer and RNA 6000 Pico Reagents Kit and 2100 Expert software (Agilent Technologies). RNA was synthesized in a thermocycler at 37°C for 7 h using MEGAscript kit (Life Technologies) and linear dsDNA primers (1–2 μg) with T7 promoter sequence (5′-TAATACGACTCACTATAGGG-3′).

### RT-PCR and RT-qPCR

Linear dsDNA was synthesized using real-time reverse transcriptase (RT) PCR in RotorGene 6000 thermocycler using Rotor-Gene SYBR Green Two-Step RT-PCR Kit (Qiagen). Adult *D. citri* extracted mRNA (250 ng) was used as template in 12.5 μL reactions. Primers were designed using Generunner software^1^ with optimal *t*_*m*_ in 50 nM NaCl of 60°C and product length of 300 bp. NCBI Blast, Flybase.org, and sohomopter.org databases were used to obtain *D. Citri* gene sequences. Thermocycler settings for the PCR were: 10 min at 95°C for the reverse transcription step followed with 40 cycles of 95°C for 5 s, 55°C for 15 s, and 72°C for 30 s. Melt was performed from 60 to 95°C with 0.2°C increments. Following PCR, the amplified DNA was purified using NucleoSpin Gel and PCR Clean-up kit (Macherey-Nagel). T7 dsDNA was amplified with Platinum PCR SuperMix (Life Technologies) and purified using NucleoSpin PCR Clean-up kit (Macherey-Nagel).

Quantitative Real-time Reverse Transcriptase (RT-qPCR) was run as was described for RT-PCR (above). The following primer set amplifying a region of the *D*. *citri* Ribosomal S20 (ew) psyllid gene (GenBank Accession # number DQ673424) was used as an internal reference to quantify psyllid cell number in each reaction: using primers: forward (5′ GCCCAAGGGCCCAATCA 3′, *t*_*m*_ 69°C) and reverse (5′ GGAGTCTTACGGGTGGTTATTCTG 3′, *t*_*m*_ 67°C) (Ammar et al., 2016). Analysis was performed using RotorGene 6000 software and the results were normalized using a *D. citri* ubiquitous ribosomal protein [RPS20 as a reference gene and ΔΔCt (Livak and Schmittgen, 2001; Wilkening and Bader, 2004; Yuan et al., 2006)]. Comparative quantitation was used to acquire representative Ct take-off points (TOP) and representative amplification efficiencies (A) for target (target) and reference genes (ref) in experimental (exp) and control (cntrl) conditions. The following formula for relative mRNA abundance was used: (A_*exp*_^–targetTOP^/A_*cntrl*_^–*targetTOP*^)/(A_*exp*_^–refTOP^/A_*cntrl*_^–*refTOP*^). A primer set flanking the RIE dsRNA subgenomic sequence, not to detect synthetic or viral RIE dsRNA, was used to determine the relative as compared with controls of Rieske mRNA abundance in total RNA of tissue extracts. These results were normalized with a ubiquitous ribosomal protein (RPS20) of *D. citri* reference gene using ΔΔCt as follows: (A_*RIE*_^–*RIECt*^/A_*cntrl*_^–*RIECt*^)/(A_*RIE*_^–*RPSCt*^/A_*cntrl*_^–*RPSCt*^), where A_*RIE*_ is the amplification of the dsRNA RIE treated sample, A_*cntrl*_ is the amplification of the diet alone sample, RIE Ct is the cycle threshold (Ct) using Rieske primers forward (5′ GGCTGATGTGTTGGCAATGG 3′, *t*_*m*_ 63°C) and reverse (5′ CTAAGTGTCTCGCGGCTGGTGTG 3′, *t*_*m*_ 52°C) and RPSCt is the Ct using RPS20 primers (Livak and Schmittgen, 2001; Wilkening and Bader, 2004; Yuan et al., 2006).

### Northern Blot Analysis

Total RNA was extracted from *D. citri* guts that were fed on CTV-wt and CTV-*RIE* infected leaves using TRIzol reagent (GIBCO BRL, Gaithersburg, MD, United States) following modified manufacturer protocol (Lee et al., 2002; Griebler et al., 2008). Denatured gut mRNA (5 μg per lane) was run by electrophoresis on 1.5% denaturing gel using Northern MAX kit (Ambion Life Technology, Austin, TX, United States), transferred to BrightStar^+^ nylon membrane (Ambion) and hybridized with a DIG-labeled 273 bp cDNA probe (Roche, Life Sciences) that was amplified by PCR from *D. citri* Rieske by using primers RIE (forward) (5′-AGCTGCTGCTGAGATTGCCAAGG-3′) and RIE (reverse) (5′-GCTCAAGGGGCTTGGACCGGA-3′) using a melt cycle at 94°C for 60 s following with 94°C for 20 s, 55°C for 20 s, and 72°C for 60 s for 30 cycles and an extension cycle of 72°C for 10 min. The blot was washed according to the manufacturer’s instructions and exposed to X-ray film for 24 h. The Rieske mRNA transfer of CTV-*RIE* and CTV-wt fed *D. citri* guts was compared with Ribosomal RNA of *D. citri* guts that were similarly treated.

### Construction of Gut Transcriptome

Whole transcriptome of *D. citri* gut cDNA library was constructed from adult *D. citri* gut mRNA (500 ng) (see RNA preparation above) that were fed on citrus leaves that were infected or not infected with engineered CTV. The purified transcript was treated with RNase III hydrolyzing dsRNA and stem loop structures and creating uniformly sized mRNAs (100–200 nt). The transcripts were hybridized for 1 h at 30°C with an adapter at the 5′PO_4_ and 3′OH sites following by reverse transcription with SuperScript^®^ III enzyme mix for 30 min at 42°C. The cDNA was then amplified by PCR using Platinum^®^ PCR SuperMix (Life Technologies) and barcode primers that are computer-identifiable and sequence specific and the PCR amplification conditions followed the manufacturer specifications (Life Technologies). Each cDNA library was constructed three times and amplified with different bar code primers. Each reverse transcription and PCR reaction was treated with magnetic bead cleanup kit (Life Technologies) and cDNA size selection (200 bp or higher) was done using 2% E-Gel (Life Technologies). The concentration and size distribution of the cDNA library was quantified using Bioanalyzer HS DNA Reagent Kit (Agilent Technologies). Libraries with average length of 150–300 bp and at concentration of 20 pM were clonal amplified and fixed on ion sphere particles (ISP) with ion one touch 2 instrument using ion PGM template OT2 200 kit (Life Technologies). Template positive ISPs were enriched with Ion ES, Ion PGM Template OT2 Solutions 200 Kit, and Dynabeads^®^ MyOne^TM^ Streptavidin C1 Beads (Life Technologies). The Dynabeads^®^ ISPs complexes were immobilized with a magnet, washed thoroughly and afterward the ISPs were melted off releasing the ISPs- cDNAs conjugates. The percentage of template positive ISPs was quantified using Qubit 2.0 Fluorimeter and Ion Sphere^TM^ quality control kit by measuring the ratio of fluorescence emission from Alexa-Fluor 488 probe-hybridized ISPs and Alexa-Fluor 647 probe-hybridized template. Samples with a ratio of relative fluorescence emission (647 nm/488 nm) of 4–50% were used following manufacturer guidelines. The amplified ISP-cDNAs were annealed with sequencing primers by melting the cDNA at 95°C for 2 min and then annealing the primers at 37°C for 2 min following by the addition of DNA sequencing polymerase and the mixture added to Ion 318 chip and sequenced using PGM sequencer (Life Technologies).

### Analysis of Gut Transcripts

cDNA libraries of adult *D. citri* guts that were isolated after feeding for 4 days on engineered CTV infected leaves were transcribed, deposited on Ion 318 chip (Ion Torrent, Life Technologies) and sequenced. More than 4 × 10^6^ reads (length 130–200 bp) were obtained after feeding *D. citri* on CTV-RIE, CTV-*gfp* and CTV-free leaves. Only high quality (>90% and containing <40% polyclonal) sequencing runs (2 – 6 × 10^6^ reads) were used. A *de novo D. citri* reference assembly was created in *Geneious 7.0* (Biomatters) using sequences from www.sohomoptera.org (Reese et al., 2014). A comparative RNA Seq analysis using QSeq and Array Star (Lasergene 11.0 suite) generated 100,517 combined reference contig hits for CTV-RIE fed and control (CTV-free) gut sequence. Moderated *t*-tests and 99% confidence intervals were used to analyze the reference hits that contained 149 contigs that were 8-fold differentially expressed showing increase or decrease between citrus leaves fed to *D. citri* expressing CTV-RIE and CTV-free leaves. No differentially expressed genes were found in gut transcriptomes of CTV-*gfp* fed and control *D. citri*.

### QBlast and Gene Ontology (GO) Analyses

**FIGURE 2:**
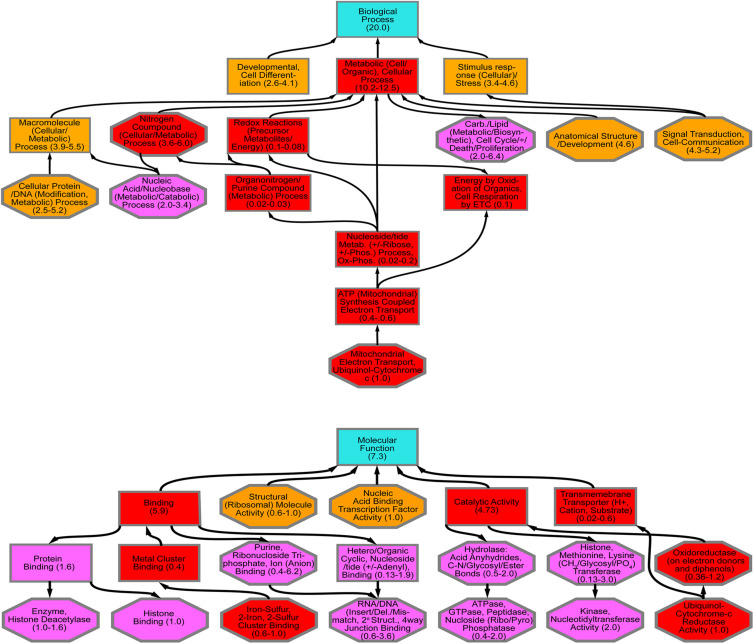
GO Annotation of comparative RNA Seq Transcriptome analysis. Contigs with the most significant variations showing up or down transcriptional abundance were identified in *D. citri* gut transcriptome after feeding on *C. macrophylla* leaves engineered with CTV-*RIE*. Using two major categories of GO Ontology (Biological Process and Molecular Function upper and lower hierarchical maps, respectively). The hierarchical maps show groups of contig specific annotations (in octagons) linked by shared functions or processes (in rectangles) each with a range of node scores in parenthesis. Each term in an octagon or rectangle is associated with a node score [Node Score = (no. of sequences) × α^*dist*^, with α = constant = 0.6, dist = a measure of the relative association to the node term]. Annotations of the contig identified as the Rieske gene are contained in the lowest octagons in each map above. Upward arrows connect to other red rectangles and octagons and represent “downstream” effects. Downward arrows (from red shapes) connect to magenta shapes that represent “upstream effects.” Orange shapes contain terms that are not (or have not yet been) associated with downstream effects of dsRNA-*RIE.*

*Diaphorina citri* contigs were analyzed by blastx against UniProt (SwissProt), NCBI (nr), and refseq_protein, databases in Blast2Go *and in flybase.org* using 1E^–6^ cut off. From 149 significant genes 25 were reliably annotated with GO terminology, showing contigs that are over and under expressed in *D. citri* gut transcriptome fed CTV-RIE (Supplementary Figure 2 and Supplementary Table 3). Blast2Go was used to generate GO ontology maps for the two GO term categories, biological process, and molecular function (Figure 2). The ontology map denotes under or over expressed contigs and shared processes or functions between related annotations. Similar annotations were grouped by node score (NS) [NS = Σ#Seqs × α (α = dist to node)] (Conesa et al., 2005; Götz et al., 2008; Figure 2).

### Data Analysis

Data were analyzed by Two-Way ANOVA to identify diets significant in mortality. Diets targeting genes with more than four significant bioassays were tested for normality by D’Agostino and Pearson’s Test followed by Two-Way ANOVA for mortality and dose response. Dunnett’s Multiple Comparison Test and One-Way ANOVA with *F*-test for equal variance, as well as Unpaired Student *t*-tests with Bartlett’s Test for variance were also used. All statistical analyses were performed using Graph pad Prism 6.0. Results were considered statistically significant when *p* < 0.05 and results are expressed as means of 6 determinations ± S.E.M., except where otherwise stated.

## Results

### Artificial Diet Bioassays

Using crowd-sourcing challenge 43 unique dsRNA sequences targeting specific *D. citri* genes were identified (Supplementary Table 1). The proposed dsRNA molecules targeted diverse groups of key biological processes in *D. citri*. In order to synthesize dsRNAs for each target homologous *D. citri* sequences were identified using genomic and transcriptomic data mining and primers were designed to amplify a 200–300 bp dsRNAs. Feeding of different concentrations of the dsRNAs (Supplementary Table 1) (48, 24, and 12 ng/μL) to psyllids were statistically analyzed. Lower dsRNA concentrations (6 and 3 ng/μL) were not significantly effective and the lowest effective dsRNA concentration is 12 ng/μL. Twenty-one diets of 43 tested dsRNAs, showed significant mortality when fed 48 ng/μL, however, only 3 dsRNA (Rieske, Cytochrome P450 4g15-like^2^ and Pterin 4α-Carbinolamine Dehydratase) showed significant mortalities at 48 ng/mL greater than 30% mortality above diet alone and significant mortalities at concentrations of 24 ng/μL and 12 ng/μL (Tables 1, 2). To find out if the mean mortalities of the three tested dsRNA concentrations that were fed to psyllids are significantly different in causing mortalities, and to find out if the mean mortalities between different pairs (48–24 ng/mL and 24–12 ng/mL) are also statistically significant. Data were analyzed by ANOVA multiple comparison tests with Fisher’s LSD (Tables 2, 3). The three most effective dsRNA targeted genes express (a) an iron-sulfur cluster subunit of the mitochondrial electron transport chain complex (Rieske), (b) a heme iron-binding terminal oxidase enzyme (Cytochrome P450), and (c) a tetrahydrobiopterin (BH_4_) pathway enzyme (Pterin 4α-Carbinolamine Dehydratase) (Tables 2, 3). In addition, three other dsRNA caused mortalities greater than 30%, only at a concentration of 48 ng/μL, they include: (d) a small molecule-ion ATP Binding Cassette Transporter subunit (ABC Transporter), (e) a ubiquitous iron binding storage protein (Ferritin), and (f) a cell adhesion calcium binding protein (E-Cadherin). Five of these six proteins (a, b, d, e, and f above) are important in cellular maintenance of iron levels (Iwata et al., 1996; Bilello et al., 2003; Mehta et al., 2009; Sztal et al., 2012; Yan et al., 2012). Sequence repeats of 3–9 base pairs that were reported in the literature in successful RNA interference studies were also identified in the dsRNAs that were used in this study. Three of these sequences, GCCC, ATGC, and TTCG were identified in the subgenomic Rieske sequence (three, two, and three times, respectively) whereas ATGC in Pterin 4α-CD once (Table 4; Elbashir et al., 2001; Yoshinari et al., 2004). The lengths of the three most effective dsRNA sequences (Rieske, Pterin 4α-CD, and Cytochrome P450) are smaller than 284 bps, and they are 10% shorter than the average length of the other 40 genes that were screened (Table 4; Lee et al., 2002).

**TABLE 1:** Statistical analysis of *D. citri* mortality of the top three dsRNAs fed by artificial diet showing dose response (Figure 1B).

Trial	Rieske Mortality ± SEM	*N*	Pterin 4α-CD Mortality ± SEM	*N*	Cytochrome P450 Mortality ± SEM	*N*
1	41.4 ± 7.6 (*p* = 2.8e^–4^)	42	29.8 ± 2.29 (*p* = 1.4e^–7^)	18	37.9 ± 11 (*p* = 6.2e^–3^)	12
2	33.3 ± 11.5 (*p* = 0.016)	42	36.1 ± 3.24 (*p* = 5.9e^–7^)	18	44.8 ± 8.63 (*p* = 4e^–4^)	12
3	37.8 ± 12.6 (*p* = 0.015)	42	N.D		N.D	

*Data was analyzed using multiple t tests (α = 0.1, Holm-Sidak corr.) comparing dsRNA diets (48 ng/μL) to diet alone. N. D. not determined. Results are considered significant when p < 0.05.*

**TABLE 2:** Statistical analysis of *D. citri* mortalities using 2-way ANOVA with Fisher’s LSD test and different concentrations the best three dsRNA diets showing dose response (Figure 1B) as compared with diet alone.

dsRNA (ng/nL)	Rieske mortality	Pterin 4α-CD mortality	Cytochrome P450 mortality
48	33.4 (*p* = 0.0001)	30.5 (*p* < 0.0001)	38.6 (*p* < 0.0001)
24	14 (*p* < 0.001)	19 (*p* < 0.001)	18.4 (*p* < 0.0001)
12	4.79 (*p* = 0.04)	1.3 (*p* = 0.66)^#^	2.66 (*p* = 0.38)^#^

*^#^Statistically not significant. Results are considered significant when p < 0.05.*

**TABLE 3:** Comparison of different dsRNA concentrations by 2-way ANOVA with Fisher’s LSD test for the three best dsRNA diets showing dose response (Figure 1B).

dsRNA pairs (ng/nL)	Rieske mortality	Pterin 4α-CD mortality	Cytochrome P450 mortality
48 and 24	19.4 (*p* < 0.0001)	11.5 (*p* = 0.08)	20.3 (*p* = 0.0013)
24 and 12	9.19 (*p* = 0.04)	20.3 (*p* = 0.002)	0.2799^#^

*^#^Statistically not significant. Results are considered significant when p < 0.05.*

**TABLE 4:** Frequency of dsRNA unique sequences.

Sequence	Rieske	Pterin 4α-CD	Cytochrome P450
GCCC	3	0	0
TTCG	3	0	0
ATGC	2	1	0
Size (bp)	273	268	270

*Each dsRNA used in this screen was analyzed for sequence repeats that have been identified through data mining of RNAi using online design tools (Life Technologies, Inc.) and size (<284 bp).*

### Leaf Bioassays

**FIGURE 3:**
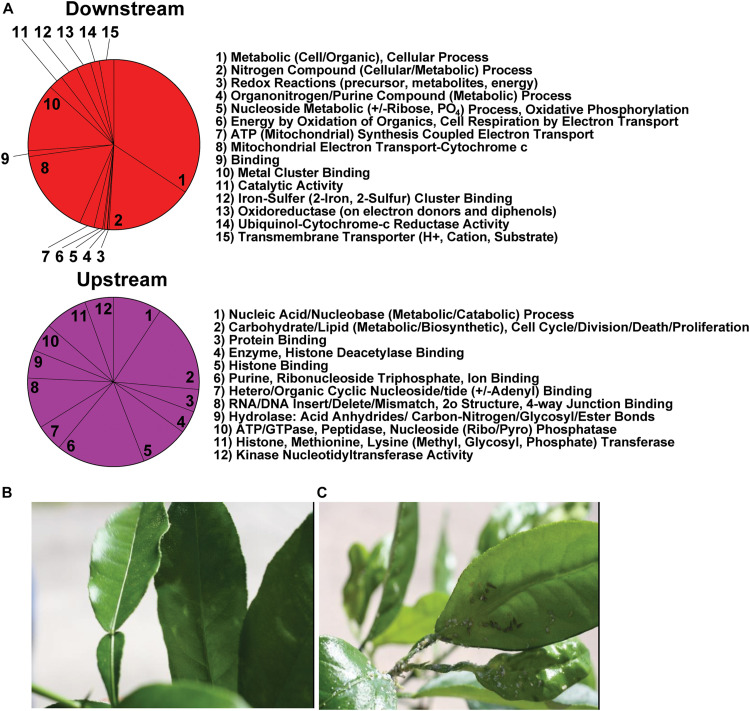
**(A)** Ontological processes arranged by upstream and downstream biological processes and unrelated effects after dsRNA-*RIE* targeting. Downstream and upstream effects that are not related specifically to down regulation of Rieske on the red and magenta pie charts include for downstream events: Nitrogen, Organonitrogen/Purine, Nucleoside and cellular and metabolic processes, and for upstream effects: RNA/DNA, and Nucleotide (Organo/Hetero) binding, metabolism, catabolism, hydrolysis, modification and repair as well as carbohydrate and lipid metabolism. **(B)** Citrus leaves infected with CTV-RIE are resistant to *D. citri* infestation, whereas **(C)** non-infected citrus leaves are not resistant to *D. citri* infestation.

The artificial diet bioassays identified genes that are affected by oral uptake of dsRNA by adult *D. citri*. Mortalities, however, never exceeded 40% after subtracting the mortalities of controls that were fed on diet without dsRNAs. Mortalities of adult *D. citri* fed on best performing dsRNAs targeting Rieske, Pterin 4α-CD and Cytochrome P450 genes, were 34%, 30.5%, and 38.6%, respectively, after subtracting mortalities caused by feeding on artificial diet alone (Figures 1A,B). Because artificial diet feeding causes mortalities of 15.7–39.1% whereas feeding on citrus leaves that expressed dsRNA-*gfp* (Supplementary Figure 1) causes very low mortalities (6%) (Figure 1C), a non-pathogenic strain of the *Citrus tristeza virus* (CTV) isolate (T36) was therefore engineered to express dsRNA in citrus leaves. The engineered virus was inoculated into *Citrus macrophylla* (*C. mac*), producing paratransgenic trees (Figure 3B) with leaves that express (in phloem ducts) subgenomic viral dsRNAs targeting *D. citri* genes that were shown to be effective in artificial diet screens.

Paratransgenic trees were created by inoculating CTV infected leaves expressing dsRNA (Satyanarayana et al., 1999). Single-detached-leaves infected with CTV expressing *D. citri* Rieske dsRNA in virus induced gene silencing (VIGS) experiments were compared with *C. Macrophylla* leaves expressing *A. victoria gfp* dsRNA (control). Because GFP is produced by CTV earlier than the *gfp* dsRNA the protein is visible in the leaves by fluorescence microscopy confirming that the plants have been successfully transfected expressing dsRNA-*gfp* (Supplementary Figure 1). Single and fully expanded young leaves from CTV engineered citrus trees were removed and the petiole placed in water filled microfuge tube (1.2 mL), sealed with parafilm, and transferred into a conical tube (50 mL) containing 15–20 adult *D. citri* then the tube was capped with a screened lid (El-Desouky et al., 2013). Adult Asian Citrus Psyllids (ACP) feeding on leaves infected with engineered CTV-RIE showed an average mortality of 76.0% whereas adults feeding on leaves that were infected with CTV-GFP showed an average mortality of 6.2% (Figure 1C). In contrast with artificial diets that causes high mortality in the absence of dsRNA only 9.5% of adult *D. citri* feeding on CTV-free leaves died (results not shown).

### RT-qPCR

**FIGURE 4:**
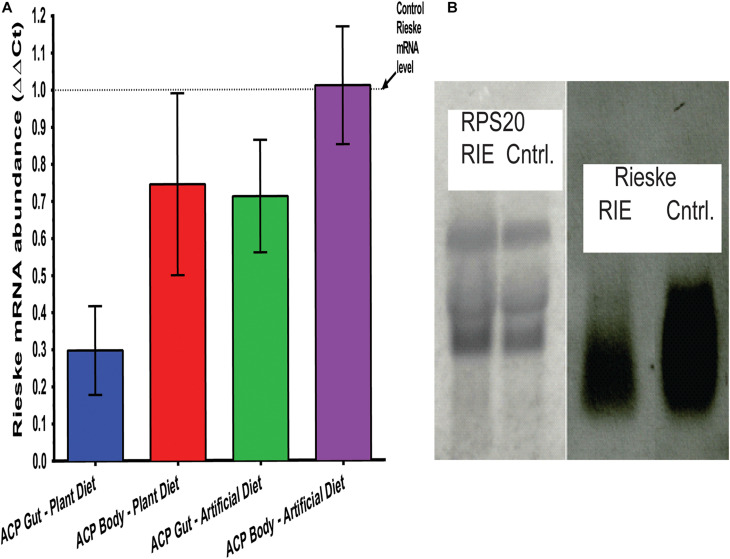
Transcriptional modification of *RIE* in *D. citri*. **(A)** mRNA-*RIE* abundance (ΔΔCt) after feeding *D. citri* on CTV-*RIE* infected citrus leaf or artificial diet containing dsRNA-*RI*E analyzed by qRT-PCR of gut and body extracts. **(B)** Northern blot analyses of control *C. macrophylla* leaf infected with CTV-*RIE* and CTV-wt (control), respectively, probed with RPS20 ribosomal proteins probe (left two lanes) and *RIE* probe (right two lanes).

RT-qPCR separately quantified the Rieske mRNA abundance in adult *D. citri* and its digestive tract. Total RNA was extracted from insects that fed on RIE artificial diets and CTV-RIE paratransgenic plants for 4 days. Analyzing adult *D. citri* in which guts were removed prior to analysis, showed that the abundance of the Rieske transcript was not affected by feeding RIE dsRNA (Figure 4A). On the other hand, gut extract transcripts that were analyzed by RT-qPCR show that feeding *D. citri* RIE dsRNA expressed in citrus leaves by engineered CTV or in artificial diet causes significant reduction in the level of Rieske transcript of 71.3 ± 3.79% and 29.8 ± 3.61%, respectively (Figure 4A). A large reduction in the RIE transcript was also observed by Northern blot analysis when gut RNA was analyzed after feeding *D. citri* on leaves infected with engineered CTV expressing RIE dsRNA as compared with insects that were fed on control leaves that were not infected (Figure 4B) or infected with *gfp*-CTV (not shown). No effect was found by Northern blot analysis when RIE dsRNA was fed and *D. citri* gut RS20 ribosomal RNA subunits were analyzed (Figure 4B). These results indicate that the Rieske transcript is affected only in the gut of the insect by feeding on artificial diet mixed with the dsRNA. If, on the other hand, the dsRNA is expressed in the leaf phloem by engineered CTV the effect is more pronounced.

### RNA Seq

Transcriptome analysis was performed on amplified mRNA extracted from the gut tissue of *D. citri* fed on CTV transformed detached leaves and sequences were assembled onto reference contigs. Of the 149 contigs showing under or overexpression of 8-fold or greater, with 99% confidence interval, after feeding *D. citri* dsRNAs expressed by CTV-*RIE* or CTV-*gfp*, 25 contigs were reliably annotated using GO terminology (Supplementary Table 1). Blast2Go generated gene ontology maps for the biological processes and the molecular functions (Figure 2). The annotated genes that were directly affected by the targeting of Rieske transcripts in these assays are found downstream (downstream effect) of the target (Rieske) gene and are colored red (Figure 2). They consist mainly of metabolic processes, cellular processes, nitrogen compound processes, mitochondria and electron transport processes and metal cluster binding (Figure 3A). Upstream effects that occur by targeting the Rieske gene or are the consequences of downstream effects were also annotated and are colored magenta (Figure 2). They consist mainly of nucleic acid processes, carbohydrate and lipid metabolism, histone binding, purine, ribonucleotide triphosphate and ion binding and RNA and DNA processes. In general, these biological processes are involved in DNA, RNA Cell Cycle, and nutrient regulation (Figure 3A). Annotated processes that were not directly attributable (given the limitations of the GO annotation database) for targeting the Rieske transcript are colored orange (Figure 2). A ranking of upstream and downstream effects on cell processes was analyzed by using the previously calculated node scores (Figure 2) allowed us to quantitate and correlate the impact of these processes with upstream and downstream effects of Rieske on *D. citri* (Figure 3A, Supplementary Figure 2 and Supplementary Table 3).

### Effect of CTV-*RIE* Infected Citrus on *D. citri*

The gene ontology studies of Rieske dsRNA with CTV-*RIE* infected citrus leaves prompted us to test the possibility that citrus trees can be protected against *D. citri*. One year old *C. macrophylla* seedlings (approximately two feet tall and stem of a pencil thickness) were infected with either CTV-*RIE* or CTV-wt control and were exposed to 100 *D. citri* adults caged in insect rearing cages (30 × 15.5 × 15 inches) and kept in growth rooms. After 3 weeks of exposure trees were observed for nymph development, adults, laid eggs, and honey-dew secretions. *C. macrophylla* plant that was infected with CTV-*RIE* is devoid of adults, nymphs, eggs or honey-dew secretions, whereas *C. macrophylla* that was infected with CTV-wt shows dense population of adults, nymphs, eggs and copious secretion of honeydew (Figure 3B) indicating that expression of Rieske dsRNA confers resistance against *D. citri.*

## Discussion

We report an innovative approach to control *D. citri* an agricultural pest insect that threatens the citrus industry in the United States and beyond. Employing a crowdsourcing strategy, we identified potentially target genes in *D. citri* that cause mortality when silenced by RNA interference. Using this information, we mined and used the *D. citri* genomics, Citrus genetics, viral engineering, molecular biology and computational biology to screen potential genes that are suitable candidates for a biological control strategy. Using gene silencing, a technique that has been shown to successfully combat insect of medical importance, as well as agricultural pest insects (Baum et al., 2007; Van Ekert et al., 2014) we rapidly (within weeks) screened potential genes for RNA interference studies using artificial diet containing dsRNA to feed adult *D. citri*. The artificial diet allowed us to rapidly monitor psyllids mortality and physiological effects. Using artificial diets to test dsRNA interference by feeding was reported for Coleoptera (*Diabrotica virgifera virgifera* and *Phylloreta striolata*) (Baum et al., 2007; Zhao et al., 2008), Diptera (*Glossina morsitans morsitans*) (Walshe et al., 2009), Hemiptera (*Acrthosiphon pisum* and *Rhodnius prolixus*) (Araujo et al., 2006; Shakesby et al., 2009), Hymenopterans (*Apis mellifera*) (Aronstein et al., 2006; Nunes and Simões, 2009), Lepidoptera (*Helicoverpa armigera*, *Pluttela xylostella*, *Spodoptera frugiperda*), and Gubernatora (Mao et al., 2007; Griebler et al., 2008; Bautista et al., 2009). The artificial diet screening identified 6 gene targets causing significant mortalities (>30%) after 6 days of feeding. Five of these genes express proteins that are directly involved or associated with proteins that maintain iron in the cell (Dhur et al., 1989; Köster, 2001; Gubernatora et al., 2003; Ana et al., 2005). These proteins include an iron-sulfur cluster domain of the mitochondrial electron transport chain (Rieske), a heme containing iron binding terminal oxidase (CP450), an iron storage protein (Ferritin), an enzyme involved in the BH_4_ biosynthetic pathway (PCD), and an ATP binding cassette transporter (ABC Transporter) indicating that cellular iron is important for *D. citri* survival.

**FIGURE 5:**
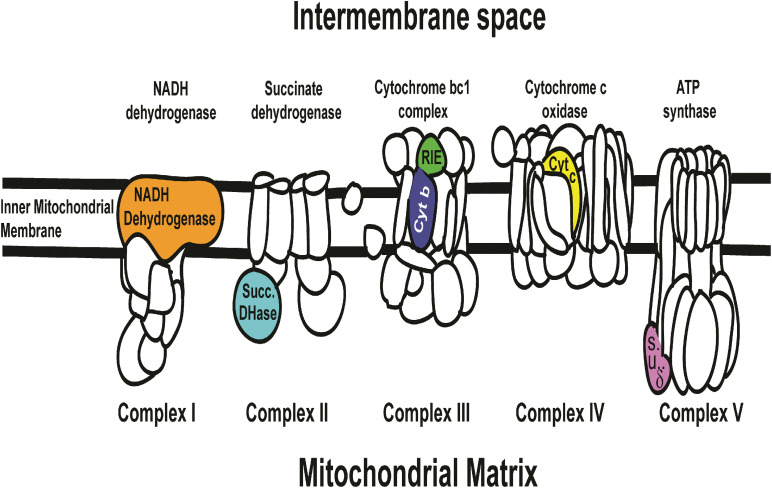
Six ETC (Electron Transport Chain complexes) genes that were screened in artificial diet bioassay (Figure 1A) in relation to the KEGG map cartoon of the oxidative phosphorylation chain in complexes I, II, III, IV, and V (NADH dehydrogenase membrane domain, Succinate dehydrogenase flavoprotein, Cytochrome bc1 complex ISP and Cytb, Cytochrome c oxidase VIa and ATP synthase δ subunit, respectively) and their relative position in relation to the intermembrane space, inter mitochondrial membrane and matrix is shown. Diets targeting Rieske (green color) and Succinate Dehydrogenase (blue color) transcripts resulted in significant mortality (see Figure 1). Cognate proteins of these two gene correspond to soluble domains of the ETC. None of the membranous domains targeted showed significant mortality (see Figure 1). Succinate Dehydrogenase (blue color) is oriented toward the mitochondrial matrix while Rieske (green color) is oriented toward the intermembrane space with greater access to the cytosolic environment. The ETC cartoon is based on available models of NADH dehydrogenase (*Thermus thermophilus*), Succinate dehydrogenase (*E. coli*), Cytochrome bc1 complex (bovine), Cytochrome c oxidase (bovine), and ATP synthase (*E. coli*). http://www.genome.jp/kegg-bin/show_pathway?map00190.

Rieske mRNA, which is translated into a soluble domain of Complex III of the Electron Transport Chain (Figure 5), significantly affected mortality by dsRNA-*RIE* (Figure 1A). Feeding *D. citri* artificial diet containing different concentrations (12, 24, and 48 ng/μL) of dsRNA-*RIE* caused a significant dose response mortality (Tables 1–3 and Figure 1B). Several reports suggest that, for every target gene an optimal concentration has to be empirically determined in order to induce optimal silencing and exceeding that optimal concentration does not necessarily result in more silencing (Liu et al., 2000; El-Desouky et al., 2013). Indeed, poor dose response relationship was found when dsRNA against CP450 was fed to *D. citri* using the same concentrations that produced good linear relationship in feeding dsRNA-*RIE* and dsRNA-*PCD* (Figure 1B). Although other genes were targeted in the electron transport chain (ETC) such as NADH Dehydrogenase, Succinate Dehydrogenase, Cytochrome b Reductase, and Cytochrome Oxidase (components of Complexes I, II, III, and IV, respectively) (Figure 5) and several showed significant mortalities. The effect was not as pronounced as observed when dsRNA-*RIE* diet was used (El-Desouky et al., 2013; Figures 1A,B). Of the five ETC transcripts screened, only the Rieske transcript is expressed in the mitochondrial intermembrane space soluble protein (ISP) of cytochrome bc1 complex. The other ETC proteins that were targeted are either membrane domains of NADH dehydrogenase, cytochrome b subunit of cytochrome bc1 complex, cytochrome c oxidase subunit VIa, or subunits located in the mitochondrial matrix like the δ subunit of ATP synthase and the Flavoprotein subunit of succinate dehydrogenase (Figure 5). The failure to affect the mitochondrial components that are part of the mitochondrial membrane or are found inside the mitochondrial matrix is perhaps due to a lower turnover of these proteins as compared with the ISP subunit (Rieske) of cytochrome bc1 complex located in the inter-membrane space. It is interesting to note that the turnover of mitochondrial proteins is generally low (6–12%) (Augustin et al., 2007). Mitochondrial proteases that are involved in mitochondrial proteins turnover are found in both the mitochondrial inter-membrane space, inner membrane and matrix, however, their activities and roles may be differently regulated (Koppen and Langer, 2007). Thus, subunits that exhibit a higher turnover are better targets for RNA silencing. Indeed, when dsRNA against Cytb, a subunit of cytochrome bc1 complex found in the inner mitochondrial membrane (Herrman and Neupert, 2003) was fed to *D.* citri no effect was observed (Figure 1A) indicating that perhaps the location of the ETC subunits in the mitochondria may determine their turnover and vulnerability to RNA silencing. Our feeding experiments did not test mixtures of dsRNAs targeting several components of the ETC. In future experiments, however, down regulating multiple components of this pathway may prove to be more effective than targeting only one transcript (Figure 1). RNA interference studies continue to highlight the complexity of insect orally induced mortality using exogenous dsRNAs. Thus, it is important to use gene internal sequences that correlate well with published and experimentally tested RNAi effects recognizing that secondary structure and base-pair length of the selected dsRNA are important in optimizing RNAi silencing effect (Elbashir et al., 2001; Lee et al., 2002; Yoshinari et al., 2004). Therefore, it is possible that designing alternate dsRNA constructs against domain genes that reside in the inter membrane space of the mitochondria like Rieske, and combinations of dsRNA targeting different genes may cause higher mortalities by oral uptake as was shown in lung cancer by reversing drug resistance (Ganesh et al., 2013) or in corneal fibroblast in which a combination of triple dsRNAs were used to target three different scarring genes (Sriram et al., 2013). In *Tribolium castaneum* 5,000 randomly screened genes were tested as RNAi potential targets and 11 genes were identified as highly efficient RNAi targets showing effective inhibition however no qPCR data or Northern blot analyses to correlate the effect of these genes with mortalities is provided. The authors similarly to our approach use GO term combinations to show the efficiency of the RNAi target genes (Ulrich et al., 2015). These genes, however, were not tested by us and in future studies we may test them even though there are not known reports that they ever were effective on the Asian Citrus psyllids.

Expressing dsRNAs in citrus using engineered CTV-*RIE* and testing feeding *D. citri* on single leaves caused 2-fold higher mortality than feeding on artificial diet containing dsRNA (65.7% and 35.4%, respectively) (Figures 1A–C), and in several cases 100% mortality was observed. These results indicate that artificial diet is inferior to feeding on engineered leaves that contain all the nutrients that are not found in artificial diet specifically when controls give very high mortality and thus these artificial diets cannot be used in long duration studies. We used artificial diets to rapidly screen several hundred dsRNA candidates that were generated by crowd sourcing to select few dsRNA candidates that were then cloned and express by engineered CTV in citrus and allowed us to test *D. citri* by feeding on engineered CTV in citrus leaves. Gong et al. (2011) reported that coating cabbage leaves with three siRNAs-*RIE* of *Plutella xylostella* caused significant mortalities lowering the Rieske transcript. Similarly, citrus leaves that were infected with CTV-*RIE* and fed to *D. citri* showed significant reduction in Rieske transcript abundance in *D. citri* gut tissue as compared with the whole insect tissue devoid of its gut. These observations support our hypothesis that oral delivery of dsRNA produces a predominantly localized response in the gut tissue and that adult *D. citri* do not amplify or transport dsRNA templates systemically as nematodes and Tribolium (Fire et al., 1998; Miller et al., 2012). The most economical way to deliver dsRNA to insects is to express dsRNA as a long hairpin (LHP) RNA in plants or cells that are eaten by insects. Baum et al. (2007) expressed LHP RNA of vacuolar ATPase (V-ATPase) in corn as an alternative to *Bacillus thuringiensis* corn expressing the *Bt* bacterial toxin and showed significant reduction in corn root damage after exposing the transgenic plants to western corn rootworm (*D. virgifera virgifera*). Following this report, two model plants *Nicotiana tabacum* and *Arabidopsis thaliana* were genetically engineered to express LHP RNA of cytochrome P450 of the cotton bollworm (*H. armigera*). Feeding this insect on leaves of the genetically transformed plants reduced cytochrome P450 transcript and larval growth was affected. Van Ekert et al. (2014) transformed yeast cells (*Pichia pastoris*) and expressed LHP-RNA against *Aedes aegypti* juvenile hormone acid methyltransferase transcript (*jmtA*). Mosquito larvae that were fed on these recombinant cells stopped larval development and the few adults that emerged died within 3 days, indicating that this technique is useful against agricultural pests, as well as disease transmitting insects. It is possible that the potency of our dsRNA-RIE could be attributed to resistance to nuclease digestion. In *Tribolium castaneum* Cao et al. (2018) reported that nucleases play a role in the stability and effectiveness of RNAi. In psyllids it was shown that dsRNA was not degraded by insect saliva that was secreted into an artificial medium during feeding. The tested dsRNA was not degraded from saliva that is excreted into the medium after feeding for 120 h (Galdeano et al., 2017). Killiny and Kishk (2017) reported that the target of the ingested dsRNA in *D. citri* is found in the gut by mixing dsRNA with a dye and microscopically showed that the dsRNA- dye was found in the psyllid gut. However, the presence of nucleases in *D. citri* gut was not tested and it is unknown what role nucleases play in digesting dsRNA in *D. citri* gut. Thus, it is important in future studies to find whether nucleases degrade dsRNA-*RIE* in *D. citri* gut. It is also possible that some of the primers that we have used were not optimal or were amplifying shorter regions that are optimal and may have to be retested in future studies.

An RNA Seq analysis of the genes affected by the dsRNA targeting the aerobic metabolic Rieske gene was performed to find out the cause of death of *D. citri* after Rieske silencing. Transcriptome analysis showed multiple potential pathways that are vulnerable after transcriptional modification in *D. citri* that could be used in future RNAi strategies (Figures 2, 3). The “downstream effects” that are not the result of Rieske or ETC malfunction include nitrogen, organonitrogen, nucleoside and nucleotide metabolic processes. Conversely, nucleic acid, nucleotide, and nucleoside binding, catalysis, phosphorylation, hydrolysis, and metabolism were identified as being indirectly affected as a consequence of the metabolic pathways of aerobic metabolism (ETC) being stressed, or “upstream effects” (Figures 2, 3). These pathways may also be sensitive to a decrease in the products of the ETC metabolism such as the oxidative state of the cell, cellular energy production, and phosphorylation. These processes are associated with cell-cycle regulation, cell-death, cell-division, cell-proliferation, DNA/RNA repair, nutrient metabolism (including carbon-nitrogen, glycosyl, peptide hydrolysis, lipid and carbohydrate biosynthesis.

Recent RNAi and enzymatic activity studies in *Drosophila* implicate an energy (or ATP) deficiency role in protein folding within the endoplasmic reticulum leading to cell-cycle arrest/delay at the G1 and G2/M checkpoints (Thomas et al., 2013). The role of cell-cycle regulation in cell-division/proliferation is well established (Zhou et al., 2002), recent studies showed that when the ER (Endoplasmic reticulum) is under stress impairment in the expression of mediators will cause cell-death, energy and nutritional deficiencies (Miller et al., 2012). The role of p53, an activator of DNA repair enzymes and mediator of cell-death (Cho et al., 1994; Xie et al., 2001), and a CHK1, a serine/threonine-protein kinase involved in cell-cycle checkpoint regulation by phosphorylation (Liu et al., 2000) in cell-death is correlated with ER stress. Thus, our virus-induced gene silencing (VIGS) is an important intermediate step in the engineering of transgenic *D. citri* resistant cultivars. Using RNA Seq analyses and next generation sequencing we identified genes (other than those targeted) that were transcriptional modified (Ratcliff et al., 1997; Ma et al., 2006; Morilla et al., 2006; Folimonov et al., 2007; Hunter et al., 2008; Velásquez et al., 2009; Gan et al., 2010; Jiang et al., 2012; Liu et al., 2013). These genes were functionally annotated, and the majority (either upstream or downstream) were found to be functionally associated with the primary function performed by the Rieske (ETC) gene targeted by VIGS (Dhur et al., 1989; Baum et al., 2007; Wuriyanghan et al., 2011; Figures 2, 3).

## Data Availability Statement

The datasets presented in this study can be found in online repositories. The names of the repository/repositories and accession number(s) can be found in the article/Supplementary Material.

## Author Contributions

JR performed the experiments, involved in the experimental design, analyzed the data, and wrote the manuscript. RJ performed the experiments. CP and WD analyzed the data and involved in the experimental design. SG performed the experiments, involved in the experimental design, and analyzed the data. DB and RS analyzed the data, involved in the experimental design, and wrote the manuscript. All authors contributed to the article and approved the submitted version.

## Conflict of Interest

The authors declare that the research was conducted in the absence of any commercial or financial relationships that could be construed as a potential conflict of interest.

## Footnotes

1 heliogenetics.com

2 https://www.ncbi.nlm.nih.gov/gene/10351

## Supplementary Material

Supplementary Figure 1 Fluorescence image (4X microscopic magnification) of leaf sample inoculated with CTV-*gfp*.

Supplementary Figure 2 **Right side**, Scatter plot of contigs matching mRNA sequenced from psyllid guts post paratransgenic CTV-*RIE* and CTV-*gfp* (control) oral uptake. Yellow contigs are equally expressed in guts from psyllid CTV-RIE or CTV-gfp paratransgenic leaves, red contigs overexpressed in CTV-RIE ACP, blue contigs overexpressed in CTV-gfp fed ACP (or under expressed in CTV-RIE fed adult Asian psyllids). The most significant (99% of contigs expression >8-fold) of under and over expression is represented by white contigs (on axes). **Left side**, 25 of 149 of these contigs have been annotated and quantified by relative RNA abundancies as read per million (RPM) showing underexpressed and overexpressed contigs (negative and positive fold changes, respectively) in CTV-RIE paratransgenic citrus fed insects.

Supplementary Table 3 Differential Gene Expression of up and down regulated genes after feeding *D. citri* CTV-RIE paratransgenic citrus.

## Abbreviations

CP450: the subgenomic fragment of the Cytochrome P450 gene
CTV: *Citrus tristeza virus*
ETC: electron transport chain
ISP: iron sulfur protein
PCD: the subgenomic fragment of the Pterin 4- α Carbinolamine Dehydratase
RIE: the subgenomic fragment of the Rieske gene
RIN: RNA integrity number
VIGS: virus induced gene silencing
wt: wild type.
