# Supplementary figures and images for: Crowdsourced Identification of Potential Target Genes for CTV Induced Gene Silencing for Controlling the Citrus Greening Vector Diaphorina citri
Source: Front Physiol. 2021 Apr 9;12:571826. doi: 10.3389/fphys.2021.571826 (PMC8063116; doi:10.3389/fphys.2021.571826)

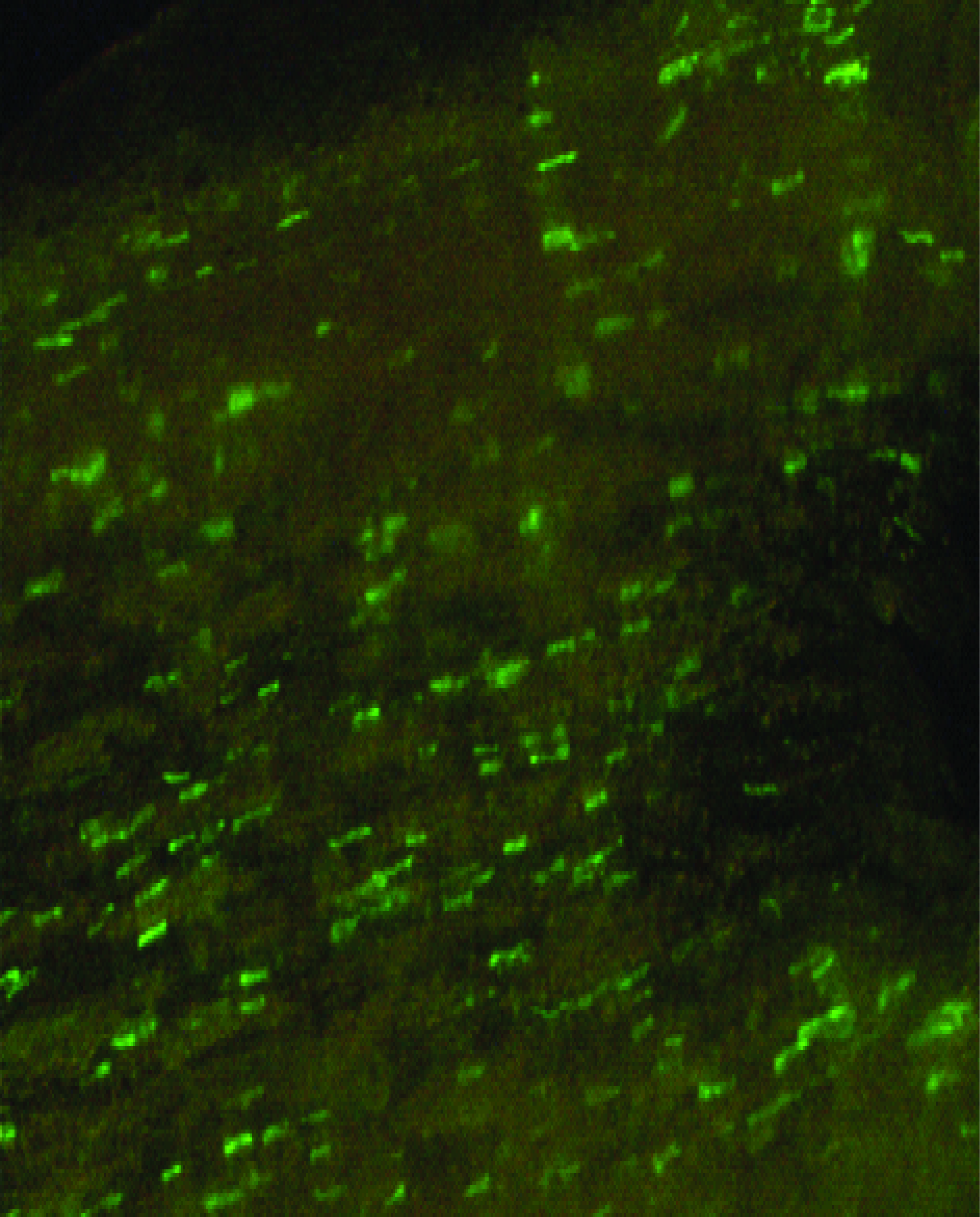

Supplement: Supplementary Figure 1 — Fluorescence image (4X microscopic magnification) of leaf sample inoculated with CTV-gfp. [file Image_1.TIF]

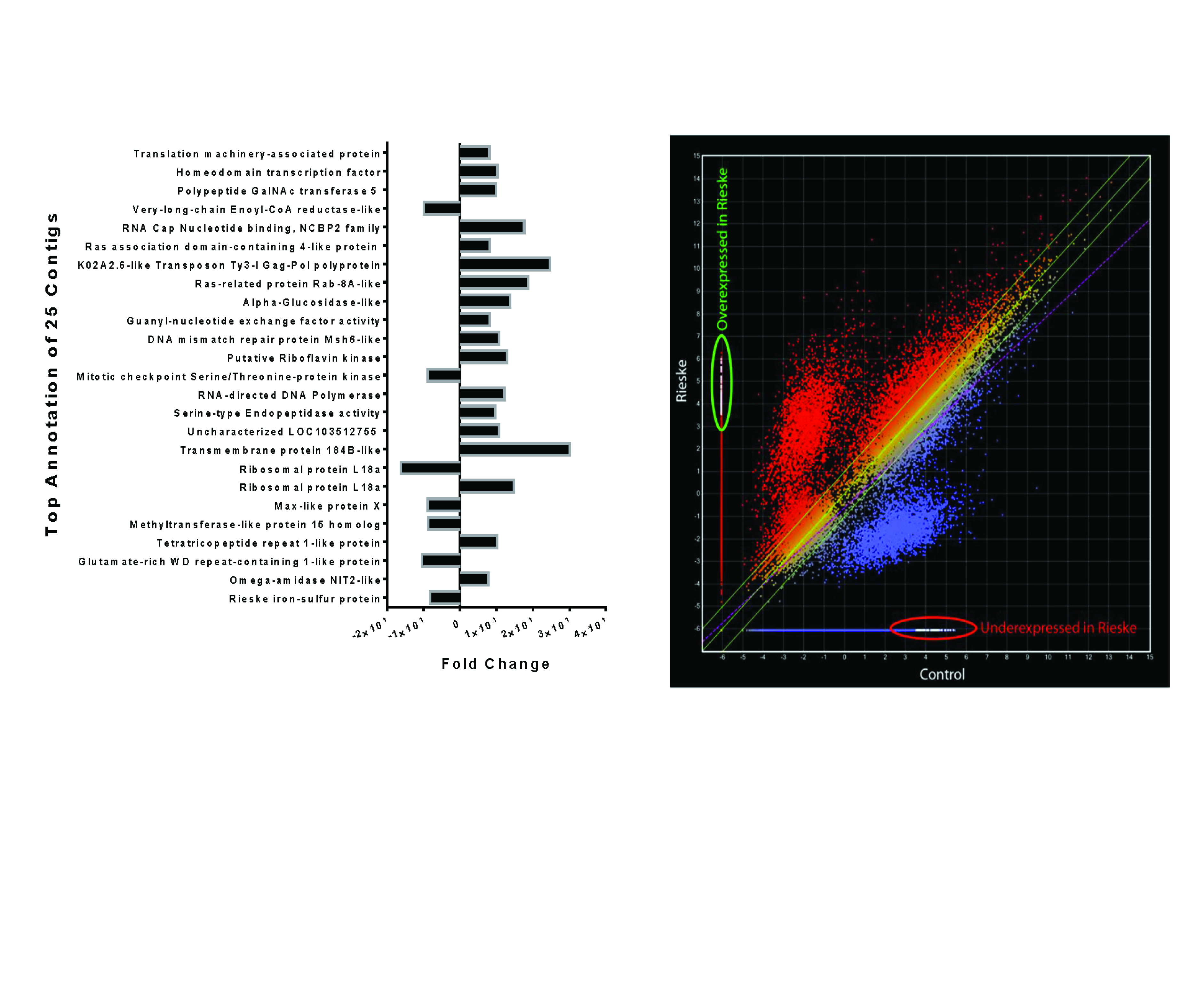

Supplement: Supplementary Figure 2 — Right side, Scatter plot of contigs matching mRNA sequenced from psyllid guts post paratransgenic CTV-RIE and CTV-gfp (control) oral uptake. Yellow contigs are equally expressed in guts from psyllid CTV-RIE or CTV-gfp paratransgenic leaves, red contigs overexpressed in CTV-RIE ACP, blue contigs overexpressed in CTV-gfp fed ACP (or under expressed in CTV-RIE fed adult Asian psyllids). The most significant (99% of contigs expression >8-fold) of under and over expression is represented by white contigs (on axes). Left side, 25 of 149 of these contigs have been annotated and quantified by relative RNA abundancies as read per million (RPM) showing underexpressed and overexpressed contigs (negative and positive fold changes, respectively) in CTV-RIE paratransgenic citrus fed insects. [file Image_2.TIF]
