# Supplementary material for: Crowdsourced Identification of Potential Target Genes for CTV Induced Gene Silencing for Controlling the Citrus Greening Vector Diaphorina citri
Source: Front Physiol. 2021 Apr 9;12:571826. doi: 10.3389/fphys.2021.571826 (PMC8063116; doi:10.3389/fphys.2021.571826)
Supplement: Supplementary file 3 [file Table_1.DOCX]

**S1 Table.** **The cognate protein names, functional grouping (parenthesis), and exact dsRNA sequences used in the 43 artificial diets.**

|  | **>Signal Transducer (Developmental-Neurological)** |  |
| --- | --- | --- |
|  | **GCCCUGAAUCAUAUCGAGUGUGCAAUUGUACCUGGCACCGUUACCUUGCAGCUGCUGUUCUCGCUUCCAUUUGAGAAGUUCGACGUUCAAUAUUUUGCCCUGUGUGUUUUCAAUGAGGAAACAUGUCUCCUUUAGAUUAUCCGCUAACUGACAGCGAUCCUGGCCUAGGGCUUUGAUCUUCGUAUUAAGAAGUUGGUCCAUCAUGAGUUUUUGCUGCUCGAAUUGUCUUUUCAGUUC** |  |
|  | **>Sugar Transporter (Transmembrane Transport)** |  |
|  | **CACCGGUCGACAAAAUGGCGGCAGAGACAAAGAUGGGCAUCAGUCAGCAGACGCUGGUCUCCAAUCAGCAGAAGGCCAAGAGAAUCACGCAGUAUAUGGCAGCUCUUACAGCCACGAUAGGCGGGUUCAUAAUGGGCACCAUCCUGGGUUGGACAUCGCCGGCGGGAGACCGCCUCAUCGCGGGCGAAUAUCCAUUUCUCGUCACCGAGUCUGACCUCUCGUUCAUCGGGAGUUCGAUGGCCCUAGGCGCCGUUUUCGGAAGCCCCGUGGUGGGGAAUCUGGUGGACACUGUGGGCCGAAAGAACACCAUGCUGCUGCUCGCGGUACCCACGCUGGUGGGGUGGGGACUGAUCAUCUGGUCUCAAUCGGUGAUCAUGUUCUGCGCUGGUAGACUCCUGACCGGGUUUGGCGGGGGCAGCUUCGCCGUGGUGGUUCCCAUGUACACGGGCCGAAUAGCCGAGACUGAAAUCCGAGGCACACUG** |  |
|  | **>Chitin Synthase (Developmental-Neurological)** |  |
|  | **GGUCUCCUCUCAUACGCCGAAAUCUGUCCUCAACUGGUCAGCAUUACCAAUGCGCAAGCAAGCCCCAGUCUACUGCGCAAACAAGAAGAUCCUCAGAGAAGAUUAGGAACGUACGCAUUCAGAUUAGCAGACAAGAAGUUGAAGCAGGAAUAUGGUACUUGGGUUAGUUUUGAGGAACCCGAGACGGCUGGAUACCAAGCGUCCUAUGCCAAGCUGGCAGGUUUGGGAGGAGUGGCUAUCAUUGAUCUGAGUCUGGAUGACUUCCGAGGCAUGUGCAAUUCCAACAAGUUCCCCAUAUUGAGAGCC** |  |
|  | **>Cytochrome P450 (Redox-Oxidative Phosphorylation Metabolism)** |  |
|  | **CAGUACACUCUCCAACCAAACUUGAAGACUGGAAGCUUCAAGCUGACAUUAUCCUCAAGAGAACAGAUGGAUUCAAAAUCCAGCUGAAACCCCGCAAGAAACAAACUGUGGCCUAACCGUUGUUACUUCUCUUUCAUUUGACCAUGUUUAUAUUUUGAUUUUUUGUACAAACUGUUUUUAUUUUUAUGGCAUUUUUCACUCCGCUUACAUGUUUUAUUUUUUUCUUUAUGACAUCCAAGUCACAUAAAGAAAUAUUGAAACUGUGAGCGG** |  |
|  | **>Amino Acid Transporter (Transmembrane Transport)** |  |
|  | **AUUUAACGGUACAUGAGAAAUCGUUCAGACAGAAGUUGGUGUUGGCAGGACUGUUUGUUAUUUCGAAUCCAAACUGCACCUCAUCCAGAAGAGUAGCUUCUUUUUCAUUGAACCAAUUCUGUUGGCAAAUCCAAUCCAGGAUGGCCUUCAAGUCAAUAGAUCCCUUCUCGCUUUGACCUACGUUGAGGAAAGAGAACACUUGAUUGACUCCGUUGGUUCCCUUAUGGACAUUCCACUUGUACCCGGCUAUUUGGACAUUGUCGUUGGUUCUACCCCCAAUAGGACCGAUCGGUCCGCAAUAUUGCACCCAGAUCAUGAUCUCAUACUUGUGCUUCUGUAACCAAAUGUCAUAAGUCAAGGAGUAGGCCAGAAGACCUUGCUUGUUAUCCACCUUGGCUCCAUAGGUAGACGUCAGGCCAUGUAUAGCAGACAAUGGCUUGUUGAUCAUAUAACCCGUAUGGGGGUAU** |  |
|  | **>ABC Transporter (Transmembrane Transport)** |  |
|  | **GUGUCAUAUCUAACAGAUGGUUGGCCGUCAGACUGGAGACUGUGGGUAAUCUGAUAACAUUCUCUGCCGCUCUGCUUGCCGUACUUAGUAAGGAGACACUCAAUGCCGGUCUGGUUGGUCUGUCAGUGAGCUACGCCAUGCAGAUCACUCAAACGCUCAACUGGCUCGUACGAAUGACGUCGGACGUGGAAACCAACAUUGUGGCCGUCGAGCGAAUCAAAGAGUACGGUGAGACCCCGCAGGAAGCCGAAUGGGUUAACAUAAGAAGCCGAAAGACGAAUGGCCAGAGA** |  |
|  | **>Oxidoreductase (Redox-Oxidative Phosphorylation Metabolism)** |  |
|  | **CCACUACUAACCACCCCACAGGAACACUUAGAAUGGGUCCUCCAUCUGAUCCUCUAGCUGUUGUUGGUCCUGACUUUAGAGUGAAUGGCUUCAGUAAUCUACGAGUUGUUGGUGAGCCUGUGAUACCUGUUGAGAUGGUUACUGACAGUAGUGCUGUUGCCCUCAUGUUGGCAGAGAGGUGUGCCACUUUCAUCCAAUCUCCCGUCAAUGUGACAACUGUCACUAAAACUACUGUAGAGAAAACUAGUGUUAUUAGUGAAGAA** |  |
|  | **>Succinate Dehydrogenase (Redox-Oxidative Phosphorylation Metabolism)** |  |
|  | **AGGAGGGAUUAGUGCUUCGUUGGGUAAUAUGUCAAAAGAUAAUUGGCAUUGGCAUAUGUUUGAUACUAUUAAAGGAUCUGAUUAUCUCGGAGAUCAAGACGCUAUUGAAUUUAUGUGCAAAGAAGCGCCAAAAGUUGUGUAUGAAUUAGAACAUUUUGGGAUGCCAUUUGAUCGAAAUAAAAAUGGUACAAUUUAUCAGAGGCCAUUUGGUGGGCAUUCAUCUAAUUUUGGAGAAAAACCUAUAGCUCGCGCUUGUGCUGUAGCUGAUCGUACCGGUCAUGCGC** |  |
|  | **>Cytochrome c1 (Redox-Oxidative Phosphorylation Metabolism)** |  |
|  | **AGGAGGGAUUAGUGCUUCGUUGGGUAAUAUGUCAAAAGAUAAUUGGCAUUGGCAUAUGUUUGAUACUAUUAAAGGAUCUGAUUAUCUCGGAGAUCAAGACGCUAUUGAAUUUAUGUGCAAAGAAGCGCCAAAAGUUGUGUAUGAAUUAGAACAUUUUGGGAUGCCAUUUGAUCGAAAUAAAAAUGGUACAAUUUAUCAGAGGCCAUUUGGUGGGCAUUCAUCUAAUUUUGGAGAAAAACCUAUAGCUCGCGCUUGUGCUGUAGCUGAUCGUACCGGUCAUGCGC** |  |
|  | **>Endoglucanase (Developmental-Neurological)** |  |
|  | **CAAGCAAGGUCUUCUGGCCUACUCCUUGACUUAUGACAUUUGGUUACAGAAGCACAAGUAUGAGAUCAUGAUCUGGGUGCAAUAUUGCGGACCGAUCGGUCCUAUUGGGGGUAGAACCAACGACAAUGUCCAAAUAGCCGGGUACAAGUGGAAUGUCCAUAAGGGAACCAACGGAGUCAAUCAAGUGUUCUCUUUCCUCAACGUAGGUCAAAGCGAGAAGGGAUCUAUUGACUUGAAGGCCAUCCUGGAUUGGAUUUGCCAACAGAAUUGGUUCAAUGAAAAAGAAGCUACUCUUCUGGAUGAGGUGCAGUUUGGAUUCG** |  |
|  | **>Heat Shock 70 (Apoptosis-Cell Cycle Regulation)** |  |
|  | **GGGUGUACUCAUCCAAGUGUACGAGGGUGAACGUGCUAUGACGAAAGACAACAACCUGUUGGGCAAGUUCGAGCUCACCUCCAUCCCACCUGCACCUCGAGGUGUGCCUCAGAUUGAAGUCACCUUCGAUAUCGAUGCUAACGGUAUCCUGAAUGUGUCUGCCAUCGAGAAGUCGACCGGUAAGGAGAACAAGAUCACCAUCACCAACGACCGUGGCCGUCUGUCCAAGGAGGACAUUGAGCGUAUGGUGAACGAUGCUGAGAAGUACAAGGCGGAGGAUGAGAAACAAAAGGCGGUCAUUACGGCUAAGAACUCCCUCGAGUCC** |  |
|  | **>Toll Receptor (Developmental-Neurological)** |  |
|  | **CGUCCGAGUGUGCCGCAAUGGCCGCCUCUGUAGCCGUGGAUUAUAGGGGUAACUGUGCCGCCCUGGGGUACAUUGGGAACUCUGCUGAGAUACAAUGCCGCAGUGACGCGAUCCAAUGUCCGCCCCUGGUGUCACCGCACUGUUUGGGUGUUACACCGCCUGGCGCUUGCUGUCCUAUUUGUGCGGGCGCGUUGAGAGUGCUUUAUAGUCAGAAACAAGUGGACAGAGCUCUGUACGCUCUCCGAGGUAGUGCCAUGUCUGCCCUCUCCGUACACGCUGUACUC** |  |
|  | **>Fascilin (Developmental-Neurological)** |  |
|  | **GGAAACCUUCAGAUGUGAUGCAGGCUAAAGUGGAGAUAGCUACAGCGACCACCAUCACUUUCUCUAUCCUGGGACCCAAUGACCCAGGAGGUAGACCUAUCAAGGCGUACUCUGUCCAAUACAAGUUCGAUUAUCAGAAUUGGAAUGAAGCAUGGAACAAGACAUGGUCUGUGGAUGCCCCUUACAUUUUGGAGAAUCUUCAGCCACAGAUGGCAUACACGUUUAGAUUUGCUGCAGUCAAUGAUGUCGGCAGUGGGAACUGGGGUGGCAUUACAUCCUACCUUAUGCCACAGAGGUCGUAUCCUGAGGAACCC** |  |
|  | **>Tropomycin (Developmental-Neurological)** |  |
|  | **GGACGCCAUCAAGAAGAAAAUGCAGGCCAUGAAAAUGGAGAAGGAUUCCGCCAUGGACAAAGCGGAUACCUGUGAGGGACAAGCUAAGGAUGCCAACCUCCGCGCUGACAAGGUACUCGAAGACGUCCGCGAAUGCCAGAAGAAGUUGACCCAGGUGGAAGUCGACUUGGACACCACCAAGAAGAAUCUCGACCAGGCCGUCAAGGAUCUGGAAGAGAGAGAAAAGGCUCUCGCCGCCGCCGAAAGCGAAGUUGCCGCCCUCAACCGCAAGGUGCAAAUGAUUGAGGAGGAUCUCGAGAAGUCCGAGGAGAGAAGUGGAACCGCC** |  |
|  | **>Cytochrome Oxidase (Redox-Oxidative Phosphorylation Metabolism)** |  |
|  | **UACAGUACACUCUCCAACCAAACUUGAAGACUGGAAGCUUCAAGCUGACAUUAUCCUCAAGAGAACAGAUGGAUUCAAAAUCCAGCUGAAACCCCGCAAGAAACAAACUGUGGCCUAACCGUUGUUACUUCUCUUUCAUUUGACCAUGUUUAUAUUUUGAUUUUUUGUACAAACUGUUUUUAUUUUUAUGGCAUUUUUCACUCCGCUUACAUGUUUUAUUUUUUUCUUUAUGACAUCCAAGUCACAUAAAGAAAUAUUGAAACUGUGAGCGG** |  |
|  | **>E-Cadherin (Developmental-Neurological)** |  |
|  | **GGAGGACCGUUCAGAUUUGAGAUGGCACCUGAAGCUUCCGAUGAUAUUCGGUCCAAGUUUAGCAUCAUUGGAGAUCAACUGAAUGCCCGCACCAUGUUUGAUCGCGAAGAGAAGAAGAUGUACCAAAUCCCCAUCGCUAUCACCGACAGUGGCAAGAACCCCAUGACUGGGACCAGCAUCCUUACUGUUAUCAUUGGAGAUCAGAAUGAUAACCCGAUGAAGUUCGGCGAGAGCUCCAUCUUUGUGUAUAACUACAAGGGAGAAAAUCCAGACACGGAAGUGGGCAGAGUAUAUGUGAAUGAUCCAGAUGACUGGGAUUUGCCCGACAAAGUGUUCAACUGGCGAGAUGCUAAGCAC** |  |
|  | **>ATP Synthase (Redox-Oxidative Phosphorylation Metabolism)** |  |
|  | **GGUCAGAGUUGACAUCCAGACUCACCGAUGCAGACCGUCCCAACUUCAACACCUUCAAAGCCAAAUACGAUGGCUACCUUCGCAAGGUGUCAGCACUUCCUGAGGCUCCACCCAAAAUUGAUUGGGCUUUGUACAAGAACAAAAUCCCCGUUCCUGGAUUAGUUGAUCAGUUCCAGAAGCAGUACGAAGCUCUUCAAAUUCCAUUCCCUCAGGACACAGAAACUGCCAAGAUCAACGAGGAAGAGAAACAGACUAUGGCUGAGAUCAAAAAAUGGAUUGAGGAAUCCCAAGUCCGCAUUGCUGGUUACAAGAAGG** |  |
|  | **>NADH Dehydrogenase (Redox-Oxidative Phosphorylation Metabolism)** |  |
|  | **GGGUGCCGGUGCUCUUGGGGCCACAUUCUACACUCUGCGACUUGCCACCAGAAACCCCGAUGUAACGUGGUUCAAGCACUCUAACCCCUACCCCUGGGAGGAGUUCAGGGAUGGCAAGCAGUACAAAUUCUACUCUCCCAACAUAGAUUACAGCAAAAUCAAAUCCCCCGCUCCCAAAUAUGAAGACUAGAUCUCUGAGUGGGAAUCUUAGAUGUUGUAUAAAAUGCUGUUAUAUUACUAUUAAGUCAUGCUAUGAACAAGACUGGGUUUGAAACCCCAAAGAUGAUAGAUCGUAGCAAAUUUUUCCCCC** |  |
|  | **>Neuroglian (Developmental-Neurological)** |  |
|  | **GGGCAAUUACGGAAAGAGUUUGAUCAUCAAACAUGUCGAGUUCUCGGACCAGGGAUCUUAUACGUGUGAAGUGAGCAAUGGAGUGGGAGAGGCCAAGAGUUACAGCAUAGACUUGUCUGUACUGGCCGCUCCUUAUUUCACUGUAGAGCCGGAGUUUAUCAACGCGGCUGAAGAAGAGACUGUUACGUUCGAGUGCAAAGCUUCUGGUCUACCAGAGCCGGAGAUCCAUUGGGUACACAACGGUAAACCCAUCUCCCAGGCACCUCCUAACCCACGCCGCAAGGUGGGACCCA** |  |
|  | **>Aquaporin (Transmembrane Transport)** |  |
|  | **GUCAUUUGAACAUUGUGCUCUCGUUUGCCUUUGCCGUGGCCACGUCUGUUAUGAUUUUCGGACACAUCAGCGGAUCUCACAUCAAUCCUGCCCUCUCAUUGGUGGGCGUAGUCAUGGGCAAAAUCUCCCUUCAGAUAUUCGUGGUGUACACCAUAGCCCAAUGCAUAGGAGCAACACUCGGCUAUUCCAUAGCAAGGUCGCUGUUUCCGGCACAUUACCUGGGCGAAACGUUCUGUUGCACUCUACCCAAUCCUAACGUUGAGCUGUCGCAAGCGUUCUCUGCAGAGUUUUUGCUGACAGUCAUCAUUGCCAUGGUCUUGUGUGCAGCUUGGGAUUACAAAUGUUUAGACAAGCACG** |  |
|  | **>Thread (Developmental-Neurological)** |  |
|  | **GGCUGAAACCAGUUACUCUCACAGAAGCUGGAUUUUUCUAUACAGGAAAGGCAGACCAGACACUUUGUUUCCGAUGUGGUGGAGGUUUGAAGCAUUGGGAAGAAACAGAUGACCCAUGGACUGAACAUGCUCGCUGGUUCUCCUCAUGUCCUUAUGUGAAGCUUGUCAAGGGUCAAGAGUUCAUCAAUCAAGUUAUUGGACACAAGGAGGUCGCCAAUGAUCCCAUUACCCUGCAGGACUUGAAAACUGUCAUCAGUGCUCACAGUGAAGUGAAACCUGCACCUAGUGUCACAGAAACUCAACCAAGCAGUAGUGCAUGCUGCACCACAGUCGCCGAGACC** |  |
|  | **>Actin (Developmental-Neurological)** |  |
|  | **CCACGAGACCGUGUACAACUCCAUCAUGAAGUGCGAUGUUGAUAUCAGAAAGGACCUGUACGCCAACACUGUCCUGUCUGGAGGUACCACCAUGUACCCCGGUAUUGCCGACAGAAUGCAAAAGGAAAUCACUGCCCUGGCUCCCUCUACCAUCAAGAUCAAGAUCAUUGCUCCCCCCGAGAGAAAGUACUCCGUGUGGAUUGG** |  |
|  | **>Fizzy (Apoptosis-Cell Cycle Regulation)** |  |
|  | **GGGAGAUGACGAGUGUGAAAUGUCCAAUUAUUAUCUGAAGAGACUUGUGACUACUGUGACACCUCCUAAGUUACUUCACACUUCCAGCGCCUCACAUACACCUAAACGUCAUCAUGCCGCCACAGAGGAAAACUCAAACACUGACCUGGACUCGAGGAAACUCUCCAAAGUUCAGAGGAAUCUCAUGACCCAUGACUUCUCCUCACACCGGAACAAACAUCUGGACCAGAACAUGAAUAUGGACCUGGAGAACGAGAAUAAUGAAAUGGAGAUGGAGAACUUAGCCACUGAGGAUGAGAAUACAGCACC** |  |
|  | **>Kayak (Transcriptional-Translational Regulation)** |  |
|  | **GGGUAUUCCGAUCAGCACGCCAUCUACCGGAAUUGUUUUCAACUUCGAAUCUCUCAUGGAGGGCGGCUCAGGAAUGACACCACUGGCAAACCCCAUCGUACCGUCUUGUUCCUCACAACAGAGAGGUGAUUGUGCCACGAGCCCCGAUGCGGUCAAUAGCAAACGCAUGUUAUCCCUGUGACAGGAGGAAUGCUGUUAGACCUGUCCAGUCUCUGCCAUUGGAGAAAUAAAGUGCCACCAACCCUUGUCCCCC** |  |
|  | **>Notch (Developmental-Neurological)** |  |
|  | **GGGUUGUAUCAAUUCCGCGUUCAAGUGUGACAAGCAGAACGACUGCGGUGAUUGGUCUGACGAGUUGGACUGCCCGUCGAAUUGUCACUACUAUAUGGCCAGCAGUGGAGAUGUCGUGGAAUCACCUAACUAUCCUCACAAGUACGAAGGUUUGGCGAACUGCAAGUGGACCCUGGAAGGACCUCAUGGACACAACAUUGUGCUGCAGUUCCAAGAGUUCGACACAGAGAAAUCAUUCGACACCGUUCAAAUCUUGGUGGGCGGCCGAGUGGAAGAGAAAUCCGUUUCCUUGGCAACCC** |  |
|  | **>Sec 61 (Transmembrane Transport)** |  |
|  | **ACCUUCAUCAUUGUCACCGCAGCCAGUACUUACCUCAUAGCAUUUGCCUACAAAAACACCAAGUUCAUCCUGAAACACAAAAUUGCUGUCAAAAGAGAAGAAUCUGUUGCCCGAGAAAUGGCCAAGAAGCUGUCAGAUGACAAAAAGAUGAGCAAGAAAGAAAAAGAUGAAAGAAUUCUGUGGAAGAAAAAUGAAGUGGCUGACUUUGAAGCCACAACCUUUUCAAUUUUCUACAACAAUGCCCUGUUCUUGGCCAUUGUUAUUUUCCUGAGUUUCUACAUCUUGAAAUCUUUCACACCUACUUUCAAUUACAUUUUCUCACUGUGGAUUUCAGCUGGAUUUUUAGCUCUGCUCUCAACUGG** |  |
|  | **>CSN 7 (Transcriptional-Translational Regulation)** |  |
|  | **GGGUGCAGCAGCUGUAGAACUCAUAAAGCAAGUACUAGAAGCUCCAGGUAUUUUCAUGUUCAAAGAGUUAAUAGACAUGCCAUUGAUCAAAGAAUUGGAUACAUCUCCUCAUGCUGGUUAUUAUCAUUUGCUGAAACUGUUUGCACAUGGAACUUAUCAGGAUUAUUUAAAACAAAAAGAAGAACUAAAACUUCCUGAGAUGACACAGCUGCAGAAGAAGAAAUUGCAAAACCUAACAAUUGUAACACUUUCAUUAGAAAGCAAAUGUAUCCCCUAUGAUAAGCUAUUGAAAGAACUUGAUAUUUCUAAUGUAAGAGAUUUGGAAGACCUUAUUAUAGAAGCCAUUUACUCAGACAUUAUCCAUGGAAAGUUGGAUCAGAGGAAUUC** |  |
|  | **>Cyclin A (Apoptosis-Cell Cycle Regulation)** |  |
|  | **GGCCCAGUAUCUGAGCGAGCUCGCCCUGGUAUCCGGGGACCCGUUCCUCCAAUUCCUCCCGUCACUCAUUGCCUGCUCCGCGAUCGCCCUGGCCCGCUACUGCCUCGACUACAAAGAGGCGUGGCCUUCAUCCCUGGCCGACAUUACCGGCCAUUCCCUCGACAGUCUCACCGAAUGUGUCAAAUGUCUGCACGAGGUACACCGCAAAGGAGAGGCCGCUAGCCAGAAGGCCGCGUACAAUAAGUAUAAACUGAACCUGUGGAAGAAUGUGUCCACCGUGGAGGCCC** |  |
|  | **>Cyclin E (Apoptosis-Cell Cycle Regulation)** |  |
|  | **GGCUUUGCAGAACAGAAGUGGAAACACCCAGGUGGAGAGAAAUCUGAGAAUGUUGAAGCAAAAGAACAUUGCACCUGGAAAUGAAAACAUUCUACCUGGGAAGAACAUUGCCACAAAUGUUUCCAAAAUACCUACCAGAAGUGUACUAGGUAUAGUAAAUGCCAAUUGUUUGCCGGACAAGAAAGCAUCAGUCAGUGACUUCAAAAAACCAUCAGCACCCUUACAACCUGUGCUCAAGAACACCAUCAGCACAAAAUUCAGUUCAACAUCCAGCCUCAACCAAAAUGUGCUACAGAACAGUUCCAGCUCUCUGAAGCCAGCUGUUCCCAGAACCAUCC** |  |
|  | **>Epidermal Growth Factor (Apoptosis-Cell Cycle Regulation)** |  |
|  | **GGGAACCCGGCCUUUGACGAUCCAAGUGUUCCCACGCAUCCCCUAUCGUUCCCCGGACUGGCUCAAUUCCUAUCGCAAGAUCCCCGCGAUCUGGCGGCCGAAUUCUCCACCAUCCCCACCACCAGUGUGCGGCCCGACGAAUUGCCGCCAGGGACGGAAGAUAAGAAUCGCUACGCCAACGUGAUCCCUAUUCCGGAGACCCGGGUUCGACUCUGCUCGGGCGGCGAAGCGGCUAGUGAUUCGGAGGAUUAUAUCAAUGCCAACUAUGUGCGGGGUCCGAAAGGUGAAGAAAAGUUCUACAUCGCAUGCCAGGCUCCC** |  |
|  | **>Polo (Apoptosis-Cell Cycle Regulation)** |  |
|  | **GGGAACUUCGAAACCUCACACUCACCCCUCACCAUCCGCUAGAAACACAUUGCUUCCUCCGUCCGCCAAGAAAAUCAAACCUCCCCUAACGGAGGAAGAAGAUUUCAAACAGCUGCUCCUACAUCGGAUAAUUCGGGACGAUGCGAAAAAUGUCGAGUUUAAAAUGGGACAGUUCUUUGGUAAGGGUGGCUUCGCAAAAUGCUACGAAGUGGUGGACCCACAGGGGCAGAUCUUCGCGGGCAAAAUCGUCUCGAAGAAGUUGUUGGUCAAGUCGAACCAACGCGAGAAGAUGAGUCAGGAGAUUGCC** |  |
|  | **>Caudal (Transcriptional-Translational Regulation)** |  |
|  | **CCACUCAUACGGACUGCGCGGUGUGUUAUUGCUUGCGUUAUACUGAUCUGUCACACUGCCAGCUGGCGGUCCGGGGGAGGCCGAAAUCUCACUGCCUGAAAUCGUAUUUGGGGUUGACGACAAGACAUGCUCUUCUUUGACAUCUUCUGAAAGCUAGGCGUGUCCGGGAACCAGUCGUUCUGGAACAUGUGGUGGGGCGGAUGGGUCCAUGUCUGGGGCUGGUCUGCCGUCUCAUGCAUACAGCUGAGGAAGGAGGG** |  |
|  | **>Osirus 7** |  |
|  | **CCAGACUGGAAAACUUCCUGAGCACACACACCCUCAAGAUUGACUUGAAAGGAAUUGAUGUGAUGAAUGCCCUCACCAGAGCUGGACGUUCCAUCAGUGACGAGGAAGAUGAAGAGGAUGUGGAAGCCAGAGGCAAACACGACAUCAUCAAGAAAAAGCACAAGAAAGGCAAGGACAUGUUGCCCCUGUUCCUCCUGUUGAAGAUGAAGUUCGCCGCUCUCCUGCCCUUCAUCCUCGGAGGAAUCGCUCUCAUCGCUGGUAAAGCCCUGCUGAUCGGUAAGAUCGCCCUGGUUCUGUCCCUGA** |  |
|  | **>Arginine Kinase (Transcriptional-Translational Regulation)** |  |
|  | **UUGGACCCCAAUGGUGAAUUCGUCAUCUCCACCCGAGUCCGUUGCGGUCGCUCCCUCCAGGGCUACCCCUUCAAUCCCUGCCUCACCGAAGCUCAAUACAAGGAGAUGGAAGAGAAGGUCUCCUCCACCCUGUCCGGACUUGAGGGAGAACUGAAGGGGCAAUUCUACCCCCUGACCGGCAUGACUAAGGAGGUGCAACAGAAGCUCAUCGAUGAUCACUUCCUGUUCAAGGAGGGAGACAGAUUCUUGCAGGCUGCCAACGCCUGCAGAUUCUGGCCCACUGGUCGCGGUAUCU** |  |
|  | **>Armadillo (Developmental-Neurological)** |  |
|  | **GGGCUUCGGACAGGGAUUCACUCAGGAUCAAGUGGAUGAAAUGAACUCCCAACUGAGCCAGACAAGAAGCCAGCGUGUCCGAGCUGCCAUGUUCCCGGAAACUUUGGAAGAAGGCAUUGAGAUUCCAUCGACUCAGUUUGACACAGCACAGCCUACUGCUGUGCAGCGAUUGACAGAGCCGAGUCAAAUGCUGAAACAUGCUGUGGUCAAUUUGAUCAACUAUCAGGAUGAUGCAGACUUGGCAACCCGUGCCAUCCCCGAGCUGAUCAAACUUCUCAACGAUGAAGAUCAAGUGGUCGUCUCGCAAGCUGCCAUGAUGGUCCAUCAGUUGUCCAAAAAGGAGGCAUCAAGACAUGCCAUCAUGAAUAGCCC** |  |
|  | **>Hopscotch (Developmental-Neurological)** |  |
|  | **GGUGGUGGAGAGUUCGGUGAUGUAUGUCGUGGUAAGCUGAAGCUCCCCCCAGACUCUCGCUCCGAUAUAGACGUCGCUAUCAAGACACUGAAACCAGGGAGUCCUGAUAAGGCACGGAAUGAUUUCCUAUCCGAGGCAUCUAUCAUGGGACAGUUCGAGCAUCCCAAUGUGAUCUUCCUACAGGGUGUCGUGACCAAGUCCAACCCUGUCAUGAUCAUCACCGAAUAUAUGGAGAAUGGGUCUCUAGACACUUUCCUGAGGGCAAAUGACGGCAAGUUCCAAGUGCUGCAACUAGUGGGUAUGUUGAGAGGUGUAGCGUCUGGUAUGC** |  |
|  | **>Nicastrin (Developmental-Neurological)** |  |
|  | **GGGUGUAGCUCGGAGAUUGAUGGCAAUGUUGGAGUACUCCAUGUUGUGGAGAGUAUGGAAGAUAUUGAUUGGCUCCUACACAAUUCAACGAGAGGGCCUUAUGUUGGUCUCCUUGAUAUUUCCAUGUUCAACAGGAGUUAUCUGGUCCCAUUGAAUUCAAGCUCAAAUAUCAAUGGAAUCAUAUUCACAUACAAUCAAACUAAUGCUGCCACCACUAAACCAAAAUUCUUCUCCCAAGAGGAUUCUUGUCCUAAUCGGUACACCUCAUUAAAUCCUCAAACCAAGCAACUGGUUUGUGAUUCAACAACACCAUGGAAUCCAUAUGGUACAAACAUAAUGAAUGAGAACUGGAACUUCCCC** |  |
|  | **>Domeless (Developmental-Neurological)** |  |
|  | **GGCCCUGGAGAACCAAGUGAACCAGUGUACAACACGACUUAUGCAUCAGCUCCUAGCAGACCCCCUGAAAUUAAAUCUGUCCAAGACAUUACCAACACAAGUGCUAAUGUCACAUGGCAGAAACCUAGUGUAGCUGAAAGAAAUGGAAACAUAGUGGAAUAUCAGAUCUGUUUCAACUCUACCAAUAGUAAUACCAUAUGUAUUACAGAUCCUGAAAAGUUUGGCUCUAGCCAGAGUGCUGUUAUAAAUAAUCUUGAUGCUUUCACUAUAUACACAGUUAAAAUUCGAGCCUUUACAAAUGCCUGGUCUGCAAAUUCAACUGGC** |  |
|  | **>Ferritin (Redox-Oxidative Phosphorylation Metabolism)** |  |
|  | **CCGACCUGGUUUUGAAGCUCUGUACAAGAAGUUAUCUGAUGAAGCUUGGGAAGAUGCUAUCGAGCUGAUCAAAUACAAUGCUAAGCGAGGCGGAAAGCUGGUGGACUUCAGUGACAUCAGAGGCCGAGACCUGAAAUCCAUGGUGGACGAGCAGAAGAAGUCGGCCAUCACCGAGUACAACUCUCUGGCCACCGCGGUAGAUCUGCAGAAGGAACUCGCGGCCGGUGCCCACGACAUUCACAAGGAGGCCAUACGUCUCGGCCAAGCCUAUCACGAUCCGG** |  |
|  | **>Puckered (Developmental-Neurological)** |  |
|  | **GGGATATGACGGAGGGGATTGTAGACATGACATGGACCTGGACTGTGATGAGGTGTATCCCAATATATTTCTCAGTGATGGAGGAACAGCCAAAAACAAGGAGTATCTGAAGAGGATTGGTATCACTCATGTCATCAATGCTGCCAAGGGGAAAAAGTTTGGGATGGTGAACACAACCAGTGATTACTACAAAGATGTGGGCATCAAATTTCTGGGCCTAGAGCTGTTGGATCTGCCCATTGCTAACATCAGCTGTCATTTCAGAGATGTGGCTGACTTCATTGAGGATGCTCTGGAAAATAATGGTAAAGTGTTAGTGCATTGTCTCATGGGCATTTCTCGTTCGAGCAC** |  |
|  | **>Salvo (Developmental-Neurological)** |  |
|  | **GCGCUCUACCGUCACUUCUCCUGUUGUUUCCGUGCUUGGUGUCCGCUCAGUUCUGGUCCUGGACAACCAACGCCCCCCGUGGAAGCACUACGUUGCGCAGUAUUCGGAACACCAGCCCGUACAAGGGUUACAGUGGAUAUGUGUUUAGUAAGGAGAGUCUGAGAGUGGCAUAUUACUAUGACCAGACGAUUGUUAUUGUGGAGGUUGGACCGAAGAGGAAGCUGCUCAACUGCGAGAUCAUC** |  |
|  | **>Survivin (Apoptosis-Cell Cycle Regulation)**  **CUGAUCCCACGUAACACGGUCAUCCCCACGAAGAAAUCCCAGAUCUUCUCGACAGCCGCUGACAACCAGAACACCGUCACCAUUCAGGUGUACGAGGGAGAGAGGCCCAUGACUAAGGACAAUCAUCUGUUGGGCAAAUUCGAUCUGACUGGAAUCCCCCCUGCACCGCGCGGUGUCCCUCAGAUUGAGGUCACCUUCGAGAUCGACGCUAAUGGUAUUCUGCAGGUAUCUGCUGAAGACAAGGGCACCGGUAACAAGGAG** |  |
|  | **>Green Fluorescence Protein (CTV-*gfp*)** |  |
|  | **ATGGCTAGCAAAGGAGAAGAACTTTTCACTGGAGTTGTCCCAATTCTTGTTGAATTAGATGGTGATGTTAATGGGCACAAATTTTCTGTCAGTGGAGAGGGTGAAGGTGATGCTACATACGGAAAGCTTACCCTTAAATTTATTTGCACTACTGGAAAACTACCTGTTCCATGGCCAACACTTGTCACTACTTTCTCTTATGGTGTTCAATGCTTTTCCCGTTATCCGGATCATATGAAACGGCATGACTTTTTCAAGAGTGCCATGCCCGAAGGTTATGTACAGGAAAGAACTATATTTTTCAAAGATGACGGGAACTACAAGACGCGTGCTGAAGTCAAGTTTGAAGGTGATACCCTTGTTAATCGTATCGAGTTAAAAGGTATTGATTTTAAAGAAGATGGAAACATTCTCGGACACAAACTCGAGTACAACTATAACTCACACAATGTATACATCACGGCAGACAAACAAAAGAATGGAATCAAAGCTAACTTCAAAATTCGCCACAACATTGAAGATGGATCCGTTCAACTAGCAGACCATTATCAACAAAATACTCCAATTGGCGATGGCCCTGTCCTTTTACCAGACAACCATTACCTGTCGACACAATCTGCCCTTTCGAAAGATCCCAACGAAAAGCGTGACCACATGGTCCTTCTTGAGTTTGTAACTGCTGCTGGGATTACACATGGCATGGATGAGCTCTACAAATAA**  **Virus primers: C-342 GTACATCGATGCGTTCTCCGGAAGAAAC (+)**  **C-1358 TTATGCGGCCGCAGGCCTTGGACCTATGTTGGCCCCCCATAG (-)** |  |
|  | **>Rieske (dsRNA-*rie*/CTV-*rie*) (redox-oxidative phosphorylation)** |  |
|  | **AGCTGCTGCTGAGATTGCCAAGGAGCAGGCAGTTGCCATCTCAACACTGAGAGATCCTCAAGCCGATAGCGATCGAGTGAAAGACCCCAAGTGGTTAGTCCTGATTGGTGTGTGCACTCATCTGGGATGTGTACCTGTTGCAAATGCTGGTGACTTCGGTGGTTACTACTGTCCATGCCATGGTTCCCATTATGATGCCTCCGGTAGAATTCGCAAGGGCCCCGCCCCTCTCAACTTGGAAGTGCCCAAATACGAGTTCCCCGAACCTGGCCT** | **AGAGGCCCAUGACUAAGGACAAUCAUCUGUUGGGCAAAUUCGAUCUGACUGGAAUCCCCCCUGCACCGCGCGGUGUCCCUCAGAUUGAGGUCACCUUCGAGAUCGACGCUAAUGGUAUUCUGCAGGUAUCUGCUGAAGACAAGGGCACCGGUAACAAGGAGAAGAUCG** |
