# Supplementary material for: Crowdsourced Identification of Potential Target Genes for CTV Induced Gene Silencing for Controlling the Citrus Greening Vector Diaphorina citri
Source: Front Physiol. 2021 Apr 9;12:571826. doi: 10.3389/fphys.2021.571826 (PMC8063116; doi:10.3389/fphys.2021.571826)
Supplement: Supplementary file 4 [file Table_2.DOCX]

|  |  |  |
| --- | --- | --- |
| **Table S2. Primers used for dsRNA synthesis** |  |  |
| **Primers sequences 5' to 3'** | Direction | *tm* ( ^0^C) |
| **Signal transducer** |  |  |
| **GCCCUGAATCATATCGAGTGTGCA** | Forward | 63 |
| **GAACTGAAAAGACAATTCGAGCA** | Backward | 69 |
| **sugar transporter** |  |  |
| **CACCGGTCGACAAAATGGCGG** | Forward | 72 |
| **CAGTGTGCCTCGGATTTCAGTCTC** | Backward | 70 |
| **Chitin Synthase (Developmental-Neurological)** |  |  |
| **GGTCTCCTCTCATACGCCGAAATC** | Forward | 69 |
| **GGCTCTCAATATGGGGAACTTGTTG** | Backward | 68 |
| **Cytochrome P450 (Redox-Oxidative Phosphorylation Metabolism)** |  |  |
| **CAGTACACTCTCCAACCAAACTTGAAG** | Forward | 67 |
| **CCGCTCACAGTTTCAATATTTCTTTATG** | Backward | 64 |
| **Amino Acid Transporter (Transmembrane Transport)** |  |  |
| **ATTTAACGGTACATGAGAAATCG** | Forward | 60 |
| **ATACCCCCATACGGGTTATATG** | Backward | 63 |
| **ABC Transporter (Transmembrane Transport)** |  |  |
| **GTGTCATATCTAACAGATGGTTGGCC** | Forward | 67 |
| **TCTCTGGCCATTCGTCTTTCGG** | Backward | 69 |
| **>Oxidoreductase (Redox-Oxidative Phosphorylation Metabolism)** |  |  |
| **CCACTACTAACCACCCCACAGG** | Forward | 70 |
| **TTCTTCACTAATAACACTAG** | Backward | 52 |
| **Succinate Dehydrogenase (Redox-Oxidative Phosphorylation Metabolism)** |  |  |
| **AGGAGGGATTAGTGCTTCGTTGGG** | Forward | 71 |
| **GCGCATGACCGGTACGATCAGC** | Backward | 73 |
| **Cytochrome c1 (Redox-Oxidative Phosphorylation Metabolism)** |  |  |
| AGGAGGGATTAGUGCTTCGTTGGG | Forward | 71 |
| **GCGCATGACCGGTACGATCAGC** | Backward | 73 |
| **Endoglucanase (Developmental-Neurological)** |  |  |
| **CAAGCAAGGTCTTCTGGCCTACTCCTT** | Forward | 72 |
| **CGAATCCAAACTGCACCTCATCCAG** | Backward | 70 |
| **Heat Shock 70 (Apoptosis-Cell Cycle Regulation)** |  |  |
| **GGGTGTACTCATCCAAGTGTACGAGG** | Forward | 71 |
| **GGACTCGAGGGAGTTCTTAGCCG** | Backward | 71 |
| **Toll Receptor (Developmental-Neurological)** |  |  |
| **CGTCCGAGTGTGCCGCAATGG** | Forward | 74 |
| **GAGTACAGCGTGTACGGAGAGGG** | Backward | 71 |
| **Fascilin (Developmental-Neurological)** |  |  |
| **GGAAACCTTCAGATGTGATGCAGGC** | Forward | 70 |
| **GGGTTCCTCAGGATACGACCTCTGTG** | Backward | 72 |
| **Tropomycin (Developmental-Neurological)** |  |  |
| **GGACGCCAUCAAGAAGAAAAUGCAG** | Forward | 69 |
| **GGCGGTTCCACTTCTCTCCTCG** | Backward | 72 |
| **Cytochrome Oxidase (Redox-Oxidative Phosphorylation Metabolism)** |  |  |
| **TACAGTACACTCTCCAACCAAAC** | Forward | 64 |
| **CCGCTCACAGTTTCAATATTTC** | Backward | 61 |
| **E-Cadherin (Developmental-Neurological)** |  |  |
| **GGAGGACCGTTCAGATTTGAGATGG** | Forward | 69 |
| **GTGCTTAGCATCTCGCCAGTTGAAC** | Backward | 70 |
| **ATP Synthase (Redox-Oxidative Phosphorylation Metabolism)** |  |  |
| **GGTCAGAGTTGACATCCAGACTCACC** | Forward | 71 |
| **CCTTCTTGTAACCAGCAATGCGG** | Backward | 69 |
| **NADH Dehydrogenase (Redox-Oxidative Phosphorylation Metabolism)** |  |  |
| **GGGTGCCGGTGCTCTTGGG** | Forward | 75 |
| **GGGGGAAAAATTTGCTACGATCTATCAT** | Backward | 67 |
| **Neuroglian (Developmental-Neurological)** |  |  |
| **GGGCAATTACGGAAAGAGTTTGATCAT** | Forward | 67 |
| **TGGGTCCCACCTTGCGGCG** | Backward | 77 |
| **Aquaporin (Transmembrane Transport)** |  |  |
| **GUCATTTGAACATTGTGC** | Forward | 56 |
| **CGTGCTTGTCTAAACATT** | Backward | 57 |
| **Thread (Developmental-Neurological)** |  |  |
| **GGCTGAAACCAGTTACTCTCACAGAAG** | Forward | 69 |
| **GGTCTCGGCGACTGTGGTGC** | Backward | 74 |
| **Actin (Developmental-Neurological)** |  |  |
| **CCACGAGACCGTGTACAACTCCATCATG** | Forward | 73 |
| **CCAATCCACACGGAGTACTTTC** | Backward | 65 |
| **Fizzy (Apoptosis-Cell Cycle Regulation)** |  |  |
| **GGGAGATGACGAGTGTGAAATGTCCAAT** | Forward | 70 |
| **GGTGCTGTATTCTCATCCTCAGTGGCT** | Backward | 72 |
| **Kayak (Transcriptional-Translational Regulation)** |  |  |
| **GGGTATTCCGATCAGCACGCCATC** | Forward | 72 |
| **GGGGGACAAGGGTTGGTGGCAC** | Backward | 76 |
| **Notch (Developmental-Neurological)** |  |  |
| **GGGTTGTATCAATTCCGCGTTCA** | Forward | 68 |
| **GGGTTGCCAAGGAAACGGATTT** | Backward | 68 |
| **Sec 61 (Transmembrane Transport)** |  |  |
| **ACCTTCATCATTGTCACCGCAGCC** | Forward | 72 |
| **CCAGTTGAGAGCAGAGCTAAAAATCCAG** | Backward | 69 |
| **CSN 7 (Transcriptional-Translational Regulation)** |  |  |
| **GGGTGCAGCAGCTGTAGAACTCATAAAG** | Forward | 71 |
| **GAATTCCTCTGATCCAACTTTCCATG** | Backward | 65 |
| **Cyclin A (Apoptosis-Cell Cycle Regulation)** |  |  |
| **GGCCCAGTATCTGAGCGAGCTCG** | Forward | 74 |
| **GGGCCTCCACGGTGGACACATT** | Backward | 75 |
| **Cyclin E (Apoptosis-Cell Cycle Regulation)** |  |  |
| **GGCTTTGCAGAACAGAAGTGGAAAC** | Forward | 69 |
| **GGATGGTTCTGGGAACAGCTGG** | Backward | 71 |
| **Epidermal Growth Factor (Apoptosis-Cell Cycle Regulation)** |  |  |
| **GGGAACCCGGCCTTTGACGA** | Forward | 73 |
| **GGGAGCCTGGCATGCGATGTAG** | Backward | 73 |
| **Polo (Apoptosis-Cell Cycle Regulation)** |  |  |
| **GGGAACTTCGAAACCTCACACTCACC** | Forward | 72 |
| **GGCAATCTCCTGACTCATCTTCTCGCG** | Backward | 73 |
| **Caudal (Transcriptional-Translational Regulation)** |  |  |
| **CCACTCATACGGACTGCGCGG** | Forward | 73 |
| **CCCTCCTTCCTCAGCTGTATGCAT** | Backward | 71 |
| **Osirus 7** |  |  |
| **CCAGACTGGAAAACTTCCTGAGCAC** | Forward | 70 |
| **TCAGGGACAGAACCAGGGCGA** | Backward | 73 |
| **Arginine Kinase (Transcriptional-Translational Regulation)** |  |  |
| **TTGGACCCCAATGGTGAATTCG** | Forward | 68 |
| **AGATACCGCGACCAGTGGGCC** | Backward | 75 |
| **Armadillo (Developmental-Neurological)** |  |  |
| **GGGCTTCGGACAGGGATTCACT** | Forward | 72 |
| **GGGCTATTCATGATGGCATGTCTTG** | Backward | 68 |
| **Hopscotch (Developmental-Neurological)** |  |  |
| **GGTGGTGGAGAGTTCGGTGATGTA** | Forward | 71 |
| **GCATACCAGACGCTACACCTCTCAA** | Backward | 70 |
| **Nicastrin (Developmental-Neurological)** |  |  |
| **GGGTGTAGCTCGGAGATTGATGG** | Forward | 70 |
| **GGGGAAGTTCCAGTTCTCATTCATTAT** | Backward | 66 |
| **Domeless (Developmental-Neurological)** |  |  |
| **GGCCCTGGAGAACCAAGTGAACC** | Forward | 73 |
| **GCCAGTTGAATTTGCAGACCAGG** | Backward | 69 |
| **Ferritin (Redox-Oxidative Phosphorylation Metabolism** |  |  |
| **CCGACCTGGTTTTGAAGCTCTGTAC** | Forward | 70 |
| **CCGGATCGTGATAGGCTTGGC** | Backward | 71 |
| **Puckered (Developmental-Neurological)** |  |  |
| **GGGATATGACGGAGGGGATTGTAGACA** | Forward | 71 |
| **GTGCTCGAACGAGAAATGCCCATG** | Backward | 71 |
| **Salvo (Developmental-Neurological)** |  |  |
| **GCGCTCTACCGTCACTTCTCCTGTT** | Forward | 73 |
| **GATGATCTCGCAGTTGAGCAGCTTC** | Backward | 70 |
| **Survivin (Apoptosis-Cell Cycle Regulation)** |  |  |
| **CTGATCCCACGTAACACGGTCATCC** | Forward | 71 |
| **CTCCTTGTTACCGGTGCC** | Backward | 66 |
| **Green Fluorescence Protein (CTV-*gfp*)** |  |  |
| **ATGGCTAGCAAAGGAGAAGAAC** | Forward | 65 |
| **TTATTTGTAGAGCTCATCCATG** | Backward | 59 |
| **Virus primers** |  |  |
| **GTACATCGATGCGTTCTCCGGAAGAAAC** | Forward (+) | 71 |
| **TTATGCGGCCGCAGGCCTTGGACCTATGTTGGCCCCCCATAG** | Backward (-) | 86 |
| **Rieske (dsRNA-*rie*/CTV-*rie*) (redox-oxidative phosphorylation)** |  |  |
| **AGCTGCTGCTGAGATTGCCAAGG** | Forward | 73 |
| **AGGCCAGGTTCGGGGAACTCG** | Backward | 75 |
